# Characterization of Tumor-Associated Macrophages and the Immune Microenvironment in Limited-Stage Neuroendocrine-High and -Low Small Cell Lung Cancer

**DOI:** 10.3390/biology10060502

**Published:** 2021-06-04

**Authors:** David Dora, Christopher Rivard, Hui Yu, Shivaun Lueke Pickard, Viktoria Laszlo, Tunde Harko, Zsolt Megyesfalvi, Elek Dinya, Csongor Gerdan, Gabor Szegvari, Fred R. Hirsch, Balazs Dome, Zoltan Lohinai

**Affiliations:** 1 Department of Anatomy, Histology and Embryology, Faculty of Medicine, Semmelweis University, 1094 Budapest, Hungary; dora.david@med.semmelweis-univ.hu; 2 Division of Medical Oncology, University of Colorado Anschutz Medical Campus, Aurora, CO 80045, USA; chris.rivard@cuanschutz.edu (C.R.); hui.yu@cuanschutz.edu (H.Y.); shivaun.luekepickard@cuanschutz.edu (S.L.P.); fred.hirsch@mssm.edu (F.R.H.); 3 Department of Tumor Biology, National Korányi Institute of Pulmonology, Piheno ut 1, 1121 Budapest, Hungary; viktoria.laszlo@meduniwien.ac.at (V.L.); harko.tunde@koranyi.hu (T.H.); megyesfalvi.zsolt@semmelweis-univ.hu (Z.M.); csgerdan@gmail.com (C.G.); szegvari.gabor@gmail.com (G.S.); 4 Department of Thoracic Surgery, Semmelweis University and National Institute of Oncology, 1122 Budapest, Hungary; 5 Division of Thoracic Surgery, Department of Surgery, Comprehensive Cancer Center, Medical University of Vienna, 1090 Vienna, Austria; 6 Institute of Digital Health Sciences, Faculty of Public Services, Semmelweis University, 1094 Budapest, Hungary; dinya.elek@public.semmelweis-univ.hu; 7 Tisch Cancer Institute, Center for Thoracic Oncology, Mount Sinai Health System, New York, NY 1190, USA

**Keywords:** SCLC, neuroendocrine subtype, macrophage, tumor microenvironment, fGSEA

## Abstract

**Simple Summary:**

To date, the therapeutic strategy and guidelines in small cell lung cancer (SCLC) are based on cancer cell-related attributes with no biomarker used in the clinical practice. In the present study, using RNAseq and IHC, we aim to characterize in the frontline the latest biomarkers of tumor-associated macrophages (TAMs), myeloid-derived suppressor cells (MDSC) and related critical elements, regulating the anti-tumor immune response. Accordingly, we extensively evaluated the TME associations in primary tumors and matched lymph node metastases in different tumor compartments (stroma and tumor nests) and neuroendocrine (NE) subtypes in limited-stage SCLC. We show the RNA gene enrichment of the most critical molecular pathways based on the Gene Ontology (GO) iteration system using thorough bioinformatics analysis to identify new molecular targets in distinct NE subtypes.

**Abstract:**

This study aims to characterize tumor-infiltrating macrophages (TAMs), myeloid-derived suppressor cells (MDSC), and the related molecular milieu regulating anti-tumor immunity in limited-stage neuroendocrine (NE)-high and NE-low small cell lung cancer. Primary tumors and matched lymph node (LN) metastases of 32 resected, early-stage SCLC patients were analyzed by immunohistochemistry (IHC) with antibodies against pan-macrophage marker CD68, M2-macrophage marker CD163, and MDSC marker CD33. Area-adjusted cell counting on TMAs showed that TAMs are the most abundant cell type in the TME, and their number in tumor nests exceeds the number of CD3 + T-cells (64% vs. 38% in NE-low and 71% vs. 18% in NE-high). Furthermore, the ratio of CD163-expressing M2-polarized TAMs in tumor nests was significantly higher in NE-low vs. NE-high tumors (70% vs. 31%). TAM density shows a strong positive correlation with CD45 and CD3 in tumor nests, but not in the stroma. fGSEA analysis on a targeted RNAseq oncological panel of 2560 genes showed that NE-high tumors exhibited increased enrichment in pathways related to cell proliferation, whereas in NE-low tumors, immune response pathways were significantly upregulated. Interestingly, we identified a subset of NE-high tumors representing an immune-oasis phenotype, but with a different gene expression profile compared to NE-low tumors. In contrast, we found that a limited subgroup of NE-low tumors is immune-deserted and express distinct cellular pathways from NE-high tumors. Furthermore, we identified potential molecular targets based on our expression data in NE-low and immune-oasis tumor subsets, including CD70, ANXA1, ITGB6, TP63, IFI27, YBX3 and CXCR2.

## 1. Introduction

One of the most recalcitrant cancers, small cell lung cancer (SCLC) is identified as a distinct disease entity with uniform therapeutic approaches and limited efficacy. Lack of understanding of the SCLC tumor microenvironment (TME) might result in limitations in therapeutic developments compared to other cancers for which many drugs are cross-validated across different cancer types recently.

There is great effort to expand immunotherapies in SCLC, however, recently for two anti-PD immunotherapies, Nivolumab’s and Pembrolizumab’s, United States Food and Drug Administration approval was withdrawn after the confirmatory phase III trials for this indication, not reaching the statistical significance for overall survival (OS). In extensive-stage disease, two randomized trials comparing etoposide-platinum doublet therapy alone to the same therapy plus a checkpoint inhibitor (atezolizumab or durvalumab) as first-line therapy showed moderate significant increases in OS (12.3–13 vs. 10.3 months) [1,2]. Importantly, these benefits are limited, and were not correlated with PD-L1 expression.

Also, conventional biomarkers used in NSCLC and other cancers such as smoking status, tumor mutation burden (TMB), and programmed cell death-ligand 1 (PD-L1) expression is not approved in SCLC clinical practice [3]. Current management of limited-stage disease includes multimodality treatment including chemotherapy and radiotherapy. Selected patients with stage I-III SCLC are eligible for curative-intent surgery [3]. Of note, despite the attempts, personalized medicine approaches have not been validated in SCLC, and there are no biomarkers that can be used for disease classification [3].

Multiple studies showed distinct tumor subsets of SCLC that can be classified into neuroendocrine (NE) subtypes based on RNA gene expression profiles clustered into specific tumor subsets [4,5,6,7]. Others showed that NE gene expression is a continuous spectrum [8]. Due to the low SCLC incidence rate, even in high volume centers, the clinical utility for no more than two distinct subtypes might be the only way for future clinical trials. According to in situ protein expression, NE-high tumors exhibit decreased CD45+ immune cell and CD3 + T-cell densities or “immune-desert” phenotype [9]. In contrast, NE-low tumors display increased immune cell densities (“immune-oasis” phenotype), especially T-cells in tumor nests [9]. Most recent data suggest that, by differential expression of ASCL1, NEUROD1 and POU2F3, or low expression of all three transcription factors with an inflamed genetic signature, four SCLC subtypes exist, with the inflamed subtype (SCLC-I) being the most sensitive to immunotherapy [10]. Others reported that a subset of NE-low SCLC tumors shows strong expression of MHC-I and is particularly responsive to STING agonism, enhancing T-cell recognition and rejection of SCLC in syngeneic murine models [11].

To date, our comprehension of crucial TME immunological mechanisms has been limited, including tumor-infiltrating lymphocytes (TILs) and tumor-associated macrophages (TAMs). TAMs represent an intriguing cell population among innate immune cells. The tumor-related activity of TAMs can vary in different tumor compartments [12,13,14]. TAMs and other inflammatory cells are attracted to the tumor stroma by releasing cytokines and chemokines produced by tumor cells [15]. In response, macrophage-released substances may prompt the migration, proliferation, and metastatic spread of cancer cells and promote angiogenesis (the “angiogenic switch”) by producing a plethora of factors [16,17]. The heavily glycosylated transmembrane glycoprotein CD68 is considered a universal marker of TAMs [18,19]

Macrophage-polarization occurs into different phenotypes: pro-inflammatory M1 or anti-inflammatory M2 macrophages. Distinct macrophage subsets might influence disease aggressiveness and survival in human tumors [12]. M1 macrophages were shown to be tumoricidal and secrete reactive oxygen species (ROS) and cytokines such as IL-6, and TNF-α, associated with microbicidal and pro-inflammatory activities [20] and express the CTLA4-receptor CD80 and the pro-inflammatory inducible nitric oxide synthase (iNOS) [19,21]. M2 (alternatively activated) macrophages express the CD163 antigen and are thought to be modulated by IL-4 and -13 and associated with tumor formation [22], secreting VEGF and TGFβ to promote angiogenesis [15,23] and establishing a pro-fibrogenic and anti-inflammatory phenotype along with connective tissue remodeling [24].

Pathological activation of relatively immature myeloid-derived suppressor cells (MDSC) with intense immunosuppressive activity is a common hallmark in malignancies promoting survival, angiogenesis, tumor cell invasion, and metastases [25,26]. CD33+ can be expressed on a variety of myeloid cells, but also serves as a reliable biomarker for MDSCs. In humans, the presence of MDSCs was reported in glioblastoma [27], pancreatic adenocarcinoma [28], breast cancer [29], and non-small cell lung cancer (NSCLC) [30].

Our current study evaluates the TME, specifically TAMs in primary tumors, and matched LN metastases according to different tumor compartments and NE subtypes. Here, we present the gene enrichment of the most critical molecular pathways based on the Gene Ontology (GO) iteration system along with thorough bioinformatics analysis to identify molecular targets in tumors with distinct NE-subtypes and infiltration-phenotypes.

## 2. Materials and Methods

### 2.1. Study Population

A total of 32 early-stage SCLC patients with available primary tumor tissue and matched LN metastases were included in our study as previously described [4,9]. All patients underwent surgical resection in the period from 1978 to 2013 at the National Koranyi Institute of Pulmonology (Budapest, Hungary). Tumor samples were histologically confirmed as SCLC by a board-certified pathologist, then, formalin-fixed, paraffin-embedded (FFPE) tissue samples were prepared from primary tumors and LN metastases immediately after surgery. Clinicopathological characteristics were referenced in our previous study [4]. From the 32 patients, 5 patients had stage IIIA and 27 patients had stage I or stage II SCLC.

### 2.2. Tissue Processing

SCLC patient tumors were fixed in 4% formalin for 2 h in RT then further fixed for 48 h in 4 °C and embedded into paraffin blocks. TMA construction from FFPE blocks was performed as previously described [31]. Briefly, an HM-315 microtome (Microm, Boise, ID, USA) was used to cut 5-micron sections from each tissue block and placed on charged glass slides (Colorfrost Plus, #22-230-890, Fisher, Racine, WI, USA). After routine H&E staining on an automated Tissue-Tek Prisma staining platform (Sukura, Osaka, Japan) a board-certified pathologist reviewed the slides and marked the tumor borders. Two 1-mm punches of tissue were taken from each donor tissue block for primary tumors, and one 1-mm punch from LN metastases blocks and seated into a recipient paraffin block in a positionally-encoded array format (MP10 1.0 mm tissue punch on a manual TMA instrument, Beecher Instruments, Sun Prairie, WI, USA).

### 2.3. Immunohistochemistry and Immunofluorescence

Five-micron sections were cut from every FFPE TMA block for IHC staining. Staining was performed on a Leica Bond RX autostainer using mouse monoclonal antibody for CD68 diluted 1:300 (ab201340), rabbit polyclonal antibody for CD163 diluted 1:400 (ab87099) and mouse monoclonal antibody for CD33 diluted 1:200 (ab11032) from abcam. Slides were stained using the Bond Polymer Refine Detection kit (#DS9800) with Leica IHC Protocol F, and exposed to epitope retrieval 1 (low pH) for twenty minutes. Clearing and dehydration of slides was performed on an automated Tissue-Tek Prisma platform and then coverslipped using a Tissue-Tek Film coverslipper.

For double IHC stainings, ImmPRESS^®^ Duet Double Staining Polymer Kit (HRP Anti-Mouse IgG-brown, AP Anti-Rabbit IgG-magenta, MP-7724, (VectorLabs, Burlingame, CA, USA) was used for secondary antibodies, and chromogenic substrates. Counterstaining was performed with hematoxylin. For double immunofluorescence, Alexa IgG anti-rabbit A488 and IgG anti-mouse A546 fluorescent secondaries were used (Invitrogen, Carlsbad, CA, USA) to visualize binding spots for epitopes CD68 and CD163. To eliminate autofluorescence on FFPE samples, TrueBlack^®^ Lipofuscin Autofluorescence Quencher (Biotium, Fremont, CA, USA) was used. The detection of protein expression was optimized in human tonsil and lung adenocarcinoma tissue as a positive control.

### 2.4. Cell Counting and Morphometry

Images of TMA sections were captured via a DP74 color CMOS camera with 10× and 20× magnification objectives in 20 MP resolution on a BX53 upright Olympus microscope. Morphometry based on stromal and tumor nest area measurements was performed by Olympus CellSens Dimensions Software package, using manual annotation of measured areas, as previously described [9]. Briefly, in the case of primary tumors, sections from two different TMA punches (A and B), in LN metastases, and a section of one TMA punch were analyzed, retrieved from different regions of resected tumors. From all TMA blocks, two separate five-micron-thick sections (with a minimum of 100-micrometer distance in Z between them) were quantified. Positive cells for immune markers CD68, CD163 were identified with manual cell counting by two independent observers using the cell counter plugin of ImageJ software [32]. CD33 expression was assessed semi-quantitatively, due to the low total numbers of cells, where 0 (0 cells), 1 (1–10 cells), 2 (11–20 cells), 3 (21 < cells) scores were given for the stroma/tumor compartment of the respective TMA core. Scoring data for CD45+ and CD3+ cellular densities and MHCII expression on tumor cells were analyzed as previously reported [9] (Tables S2 and S3). Square micrometers (μm^2^) were converted to square millimeters (mm^2^) for the calculation of cell density parameters in statistical analyses. Results (cell numbers and areas) from separate sections of the same TMA punches were averaged for statistical assessment.

### 2.5. Cut-Off Values and Tumor Classification

We identified cut-offs for cellular density parameters and expression scores (CD68s, CD68t, CD163s, CD163t, CD33s and MHCII immune, MHCII tumor, CD45s, CD45t, CD3s, CD3t obtained from our previous study) with the median absolute deviation (MAD) method. Cut-off values and distribution of HIGH (2–3) and LOW (0–1) groups, according to NE subtypes, are shown in Tables S2 and S3 and Figure S2. HIGH vs. LOW annotations for individual samples are shown in Table S4. Based on cellular densities and expression scores of biomarkers CD68, CD163, CD33, CD45, CD3, we classified tumor samples into synthetical categories. Immune-oasis tumors (*n* = 25) have to be classified as HIGH for either stromal or intratumoral CD45+ cell density and HIGH for either stromal or intratumoral CD3+ cell density, whereas immune-desert (*n* = 32) tumors have to be classified as LOW for both stromal and intratumoral CD3+ cell density and LOW for either stromal or intratumoral CD45+ cell density (Figure S9).

### 2.6. Molecular Analysis

RNA expression data from primary and LN FFPE tumor tissue samples were obtained using the HTG EdgeSeq Targeted Oncology Biomarker Panel of 2560 cancer-related genes. Patient samples were clustered into NE-low and NE-high subtypes according to their neuroendocrine gene expression pattern, as previously reported [4,6]. The assay was validated using negative and positive process controls. All samples were run as singletons.

### 2.7. Data Pre-Processing and fGSEA Pathway Analysis

Both data pre-processing and cluster analysis were performed with R packages, including ggplot2 and *ComplexHeatmap* [33] (R package version 2.6.2) for plotting heat maps. fGSEA and gene pathway analysis were performed according to the Gene Ontology (GO) iteration system with *data.table* and *fGSEA* (1.13.2). Volcano plots and differential gene expression panels were compiled with *ggrepel* (0.8.2), *limma* (3.46.0) and *EnhancedVolcano* (1.8.0) R packages. String maps were generated with the Cytoscape Software Package (3.8.2) and with R package *RCy3* (2.10.2). In string maps, nodes are red in molecular networks of NE-low and immune-oasis tumor subsets and are blue in molecular networks of NE-high and immune-desert tumor subsets, where it is applicable. Darker color shade and larger node size represent higher LogFC values, thicker and darker edges represent stronger connection among two nodes. Connection strength is determined by an aggregate molecular interaction score (Cytoscape). Networks are organized according to the spring-embedded layout and based on a “force-directed” paradigm. Only genes with *p* < 0.05 and Log2 FC > 1.5 values are indicated in string maps. Occurrence-matrices and Venn-diagrams were generated with *UpSetR* (1.4.0) R packages.

### 2.8. Cell Line Data

Protein expression data of NE subtype markers (ASCL1, NEUROD1, YAP1 and POU2F3) and of NE-low and NE-high targets were obtained from the *Expression Atlas* and the *Cancer Cell Line Encyclopedia* [34]. Relative expression values were available for *n* = 28 cell lines, where those showing strong expression of YAP1 (*n* = 5) or POU2F3 (*n* = 3) are classified as NE-low and those with strong expression of ASCL1 (*n* = 12), NEUROD1 (*n* = 6), or both (*n* = 2) are classified as NE-high [5].

### 2.9. Statistical Methods

First, using the Kolmogorov–Smirnov test, we determined that no variable follows a normal distribution. Next, to test whether the population mean ranks differ between core A and B of the same patient, we used the Wilcoxon Matched-Pairs Signed Ranks Test, where we found no significant differences regarding any variables. Accordingly, we used averaged core A and B values in further statistical analyses. Spearman’s rank correlation (r_s_) and Kendall’s Tau-b were used to compare macrophage cell densities (CD68, CD163, and CD33) and cell densities of CD45+ immune cells, CD3 + T-lymphocytes and MHCII tumor cell expression scores, published earlier [9] on the same cohort. The correlation coefficient (r_s_) can vary between −1 to 1. We define the degree of correlation on the basis of the recommended reference [35]. We used the Mann–Whitney U test to compare CD68, CD163, and CD33 expressions between primary tumors and LN metastases, and between NE-low and NE-high subtypes in the stroma or tumor compartments. *P*-values less than 0.05 indicate the significance, and all *p*-values were two-sided.

## 3. Results

### 3.1. The Phenotype of Tumor-Associated Macrophages and Their Tissue Distribution According to SCLC NE Subtypes

**Figure 1:**
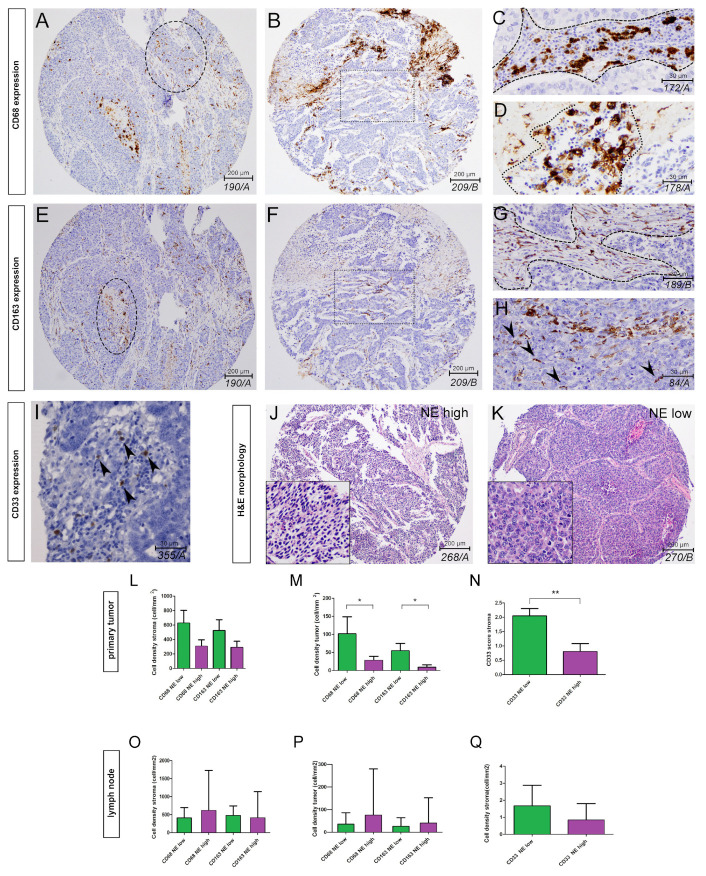
Expression of macrophage markers in SCLC tissue samples according to neuroendocrine (NE) subtypes. Immunohistochemistry for CD68, CD163 and CD33 in FFPE TMA samples. CD68 is expressed in cells of various morphologies, including amoeboid and ramified (**A**–**D**). CD68+ macrophages occur mostly in stromal bands (**C** dashed line) and areas of necrosis (**D** dashes line). In heavily infiltrated tumors, CD68+ macrophages are also present in tumor nests (**A** encircled area). CD163 is expressed mostly in ramified cells, with long, slender processes with high density in the stroma compartment (**F** boxed area, **G**) and sparsely in tumor nests (**H** arrowheads). CD163 is also expressed in macrophages populating necrotic areas (**E** encircled area). CD33 shows notable expression only in the stroma compartment on a low number of myeloid cells (**I** arrows). Stainings show the characteristic histology of NE-high (**J**) and NE-low tumor subtypes (**K**). In primary tumors (**L**–**N**), there were no significant difference regarding CD68+ cellular densities (625 ± 182 vs. 305 ± 91 cell/mm^2^, *p* = 0.065, *n* = 29; (**L**) and CD163+ cellular densities (523 ± 150 vs. 290 ± 86 cell/mm^2^, *p* = 0.16, *n* = 29; (**L**) in NE-low compared to NE-high tumors in the stroma compartment. However there was a significant increase in CD68+ (101 ± 47 vs. 28 ± 10 cell/mm^2^, *p* = 0.048, *n* = 29; (**M**) and CD163+ (54 ± 20 vs. 9 ± 7 cell/mm^2^, *p* = 0.024, *n* = 29; (**M**) cellular densities in tumor nests of NE-low compared to those of NE-high tumors. CD33+ myeloid cell density was significantly higher in NE-low compared to NE-high tumors (2.05 ± 0.3 vs. 0.8 ± 0.3, *p* = 0.006, *n* = 29; (**N**). In lymph node metastases (**O**–**Q**) there were no statistically significant differences in terms of CD68+ (stroma, 410 ± 234 vs. 614 ± 1045 cell/mm^2^, *p* = 0.156, *n* = 29, (**O)**; tumor, 36 ± 50 vs. 76 ± 197 cell/mm^2^, *p* = 0.312, *n* = 29, (**P**) and CD163+ (stroma: 496 ± 245 vs. 410 ± 805 cell/mm^2^, *p* = 0.217, *n* = 29, (**O)**; tumor, 29 ± 51 vs. 46 ± 102 cell/mm^2^, *p* = 0.187, *n* = 29, (**P**) cellular densities between NE-low and NE-high tumors, neither in the stroma nor in tumor nests (tumor). CD33+ cellular density was increased in NE-low compared to in NE-high tumors, but the difference did not reach statistical significance (1.8 ± 1.1 vs. 0.9 ± 1, *p* = 0.061, *n* = 29; (**Q**). *p* < 0.05 considered significant: * *p* < 0.05 ** *p* < 0.01 *** *p* < 0.001.

IHC shows that CD68 is expressed either in the tumor or the stroma compartment, and exhibits amoeboid or ramified morphology (Figure 1A–D), whereas CD163+ cells display ramified morphology in the same localizations (Figure 1E–H). CD68+ cells with amoeboid morphology frequently occur in areas of necrosis (Figure 1D). Expression of CD33 is lower compared to CD68 or CD163, and occurs exclusively in the stroma compartment (Figure 1I). Primary tumors and LN metastases were clustered into NE-low (primary *n* = 11, LN *n* = 8) and NE-high (primary *n* = 20, LN *n = 24*) subtypes according to their NE gene expression patterns, as previously reported [4,5]. H&E stained samples show the distinct morphology of a typical NE-high and NE-low tumor (Figure 1J,K). There was no statistically significant difference in CD68+ and CD163+ cell densities between the stroma of NE-high and NE-low primary tumors (Figure 1L) or LN metastases (Figure 1O) stroma. In tumor nests, NE-low compared to NE-high tumors were associated with significantly increased densities of CD68+ (*p* = 0.048) and CD163+ cells (*p* = 0.024) in primary tumors (Figure 1M), but not in LN metastases (Figure 1P). CD33+ myeloid cells showed significantly higher cell densities in NE-low than in NE-high primary tumors (*p* = 0.006; Figure 1N), but not in the case of LN metastases (Figure 1Q).

Next, to further analyze the spatial co-occurrence and potential interactions of TAMs and other common immune cells, we correlated the expression of macrophage markers in primary tumors (Table S1A). Stromal and intratumoral expression showed a significant positive correlation for CD68 (r = 0.845) and a significant moderate positive correlation for CD163 (r = 0.594). The correlation between CD68 and CD163 expression was significant and strong in tumor nests (r = 0.764) and moderate in the stroma (r = 0.455) CD33 expression showed significant correlation only with CD163 (r = 0.651 in stroma and r = 0.605 in tumor nests). Correlation coefficients in LN samples are shown in Table S1B.

Afterward, we analyzed the associations of macrophages’ cellular densities with the expression of pan-leukocyte marker CD45, T-cell marker CD3, and major histocompatibility complex II (MHCII) on tumor cells. Regression analysis showed a significant strong positive correlation between the cellular densities of CD3 + T-cells and CD68 + TAMs (r = 0.812) and CD163 + M2-polarised TAMs (r = 0.707) in tumor nests (Figure S1A,B). In the stroma, CD3+ cell density showed remarkable correlation exclusively with CD163+ cell density (r = 0.864; Figure S1C). The latter might implicate that lymphocytic infiltration associated with CD68 + TAM colonization is specific for tumor nests and the two processes are more independent from each other in the stroma compartment, where M2-TAM occurrence depends rather on T-cell density. The intratumoral density of CD45+ immune cells (r = 0.753), CD3 + T-cells (r = 0.705), and CD163 + TAMs (r = 0.801) all showed a significant strong positive correlation with MHCII expression in tumor cells (Figure S1D–F), meaning that both TAM and TIL colonization are coexistent with MHCII expression of tumor cells.

**Figure 2:**
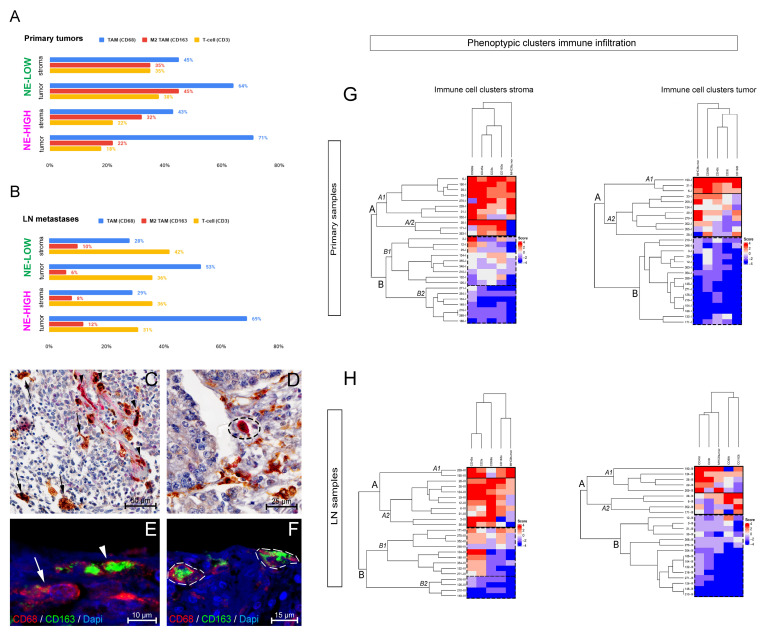
Cluster-analysis of immune phenotypes and cellular composition of the stroma and tumor compartment. Bar charts show the composition of CD45+ immune cells, including CD68 + TAMs, CD163 + M2-TAMs and CD3 + T-cells in the stroma and tumor compartment of primary tumors (**A**) and LN metastases (**B**). Double stainings show coexpression of CD68 and CD163 on TMA specimen. CD68 is labelled by brown chromogen DAB, CD163 was developed by VectaRed. IHC shows CD68 + CD163- TAMs (**C** arrows) and CD68 + CD163+ M2-polarised TAMs (**C** arrowhead, **D** encircled area) in stromal band and tumor nest. Double immunofluorescence shows CD68 expression in TAM (**E** arrow) and CD68/CD163 colocalization in double positive M2-TAMs (**E** arrowhead, **F** encircled areas). Heatmaps show different clusters of SCLC primary (**G**) and metastatic LN tumors (**H**) based on immune cell densities and MHCII protein expression. Clusters are created according to macrophage marker expression and their comparison with CD45+ immune cell and CD3 + T-cell densities separately in the tumor stroma and tumor nests.

Subsequently, we analyzed the stromas’ cellular composition and the tumor compartment separately in NE-low and NE-high tumors. CD68 + TAMs make up 64% (NE-low) and 71% (NE-high) of CD45+ cells in tumor nests of primary tumors, while T-cells only represent 38% (NE-low) and 18% (NE-high) of immune cells. CD163 + M2 macrophages constitute 45% (NE-low) and 22% (NE-high) of the immune cell pool in tumor nests. Interestingly, there were no significant differences in TAM and T-cell ratios between NE-low and NE-high tumors in the stroma compartment (Figure 2A). Results are similar in LN metastases with significantly lower overall CD163 + TAM ratios and higher CD3 + T-cell infiltrations, especially in the NE-high phenotype (Figure 2B), whereas Figure 2C–F shows the co-expression of CD68 and CD163 with double IHC and immunofluorescence on TAMs.

Heatmaps show clusters of tumors based on macrophage and lymphocyte infiltration using cut-offs described in Tables S2 and S3. In addition to the cluster of “macrophage-high” and “macrophage-low” tumors, a subset of tumors shows increased expression of CD68 and CD163 in the stroma, but low expression of CD163 in tumor nests and MHCII on tumor cells (Figure 2G). In LN metastases, we identified a subset of tumors with increased stromal CD45+ immune cell and CD3 + T-cell densities, but low stromal macrophage density and MHCII expression on tumor cells (Figure 2H). On the contrary, in tumor nests, a macrophage-high, but CD45- and CD3-low subset of tumors occurs that does not show up in primary tumors only specific to LN metastases (Figure 2H).

### 3.2. fGSEA Pathway Analysis in Different Subsets of Tumors According to NE Phenotype and Macrophage-Infiltration

**Figure 3:**
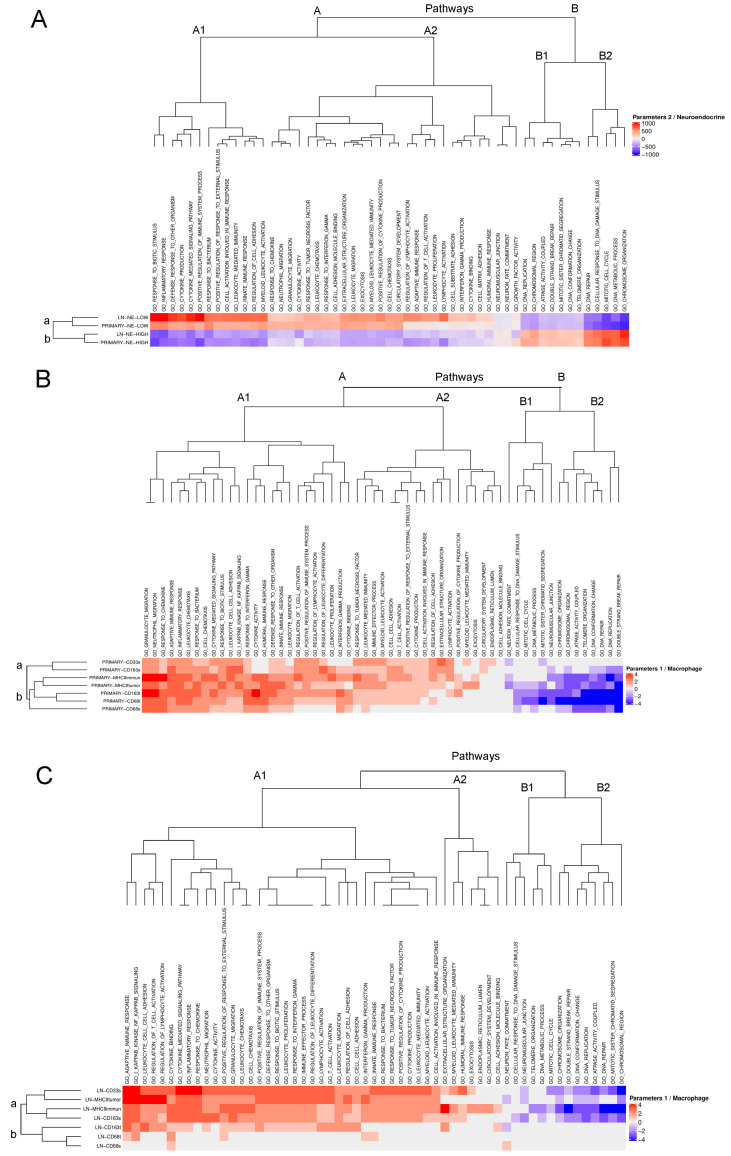
Cluster analysis on fGSEA pathways relevant in SCLC. Heatmaps show relevant Gene Ontology (GO) pathways revealed by fGSEA analysis of primary tumor and LN metastases from SCLC patients. Pathways with significant normalized enrichment scores were included (NES > 1). Every row represents HIGH tumors pooled for the given biomarker or the corresponding NE-phenotype (NE-low vs. NE-high). Every column represents a pathway whose z-score derived from the pathway’s NES score. NES scores are calculated based on the relative expression (HTG Edge RNAseq panel) of genes in corresponding pathways. (**A**) According to NE phenotype (high vs. low), two main clusters are revealed: in group A pathways related to inflammatory response, cytokine-mediated activities and pathways relevant to innate immune response, especially in *myeloid leukocyte activation*, are present. In cluster B, cell proliferation pathways, including *mitotic cell cycle*, *DNA repair*, *DNA replication*, and *chromosome organization* are displayed. Pathways in cluster A show upregulation in NE-low tumors, whereas Cluster B pathways are upregulated in NE-high tumors. As expected based on in vitro data, pathways show a strong negative correlation with each other between different NE phenotypes. Pathway up- and downregulation in the same cluster shows significant positive correlation between primary tumors and LN metastases within the same NE phenotype. (**B**) Tumors classified as HIGH for macrophage markers (CD68, CD163, CD33), MHCII immune cell and tumor cell expression (MHCII immune, MHCII tumor) show a great level of overlap in up-and down-regulated pathways. Expectedly, pathways of an i*nflammatory response* and *innate immunity* were clustered together in subcluster A1. Pathways related to adaptive immunity were clustered in subcluster A2 in a very similar way to NE-low tumors. (**C**) Cluster analysis showed no significant difference in the scope of up- and downregulated pathways or in average NES scores in LN metastases compared to primary tumors, except for minor alterations. In LN metastases MHCII-HIGH (both immune and tumor cell) tumors were clustered together, with CD33- and CD163 stroma-HIGH tumors showing strong up- and downregulation pathway profiles, both in cluster A and B. Contrary to primary tumors, CD68 expression (both in stroma and tumor nests, cluster B) is not as relevant in LN metastasis as it is in primary tumors.

Next, fGSEA analysis was performed on the HTG EdgeSeq Targeted Oncology Biomarker Panel of 2560 cancer-related genes, in order to analyze the most critical pathways using the Gene Ontology (GO) database. Heatmaps show enrichment scores (NES) of GO pathways in primary tumors (Figure 3B) and LN metastases (Figure 3C) according to NE-low and NE-high phenotype (Figure 3A), CD68+, CD163+, CD33+ cellular densities and MHCII-expression in tumor cells (in brief: macrophage-related parameters). IDs and high vs. low annotations for patient samples are shown in Table S4.

NE-high tumors showed increased enrichment in pathways related to oncogenesis and cell proliferation (Figure 3A, cluster B). On the contrary, pathways of immune response were significantly downregulated (Figure 3A, cluster A). In NE-low tumors, however, oncogenic pathways showed negative NES scores (Figure 3A, cluster B), while pathways of immunological activity were significantly upregulated. Interestingly, pathways not directly related to immune function, such as vascular system development (angiogenesis) and extracellular matrix organization, were also upregulated in NE-low tumors (Figure 3A).

Tumors with increased macrophage-related parameters displayed a significant overlap in up-and downregulated pathways with NE-low tumors (Figure 3A,B). Although tumors showing increased macrophage-related parameters displayed an apparent homogeneity of pathway enrichment profiles in primary tumors (Figure 3B), high CD68 + TAM density in LN metastases proved to be less relevant in determining the overall pathway enrichment profile (Figure 3C). Other enrichment scores for primary and LN metastases show a strong correlation in corresponding pathways supporting the fact that matched primary tumors and LN metastases exhibit similar molecular patterns and similar immunological and NE phenotypes. Figure S3 shows data for all relevant GO pathways in different tumor subsets, including NESs, fGSEA plots, and *p*-values.

### 3.3. Detailed Analysis of Representative “Oncogenic” and “Immune Response” Pathways Related to the Gene Ontology (GO) System

**Figure 4:**
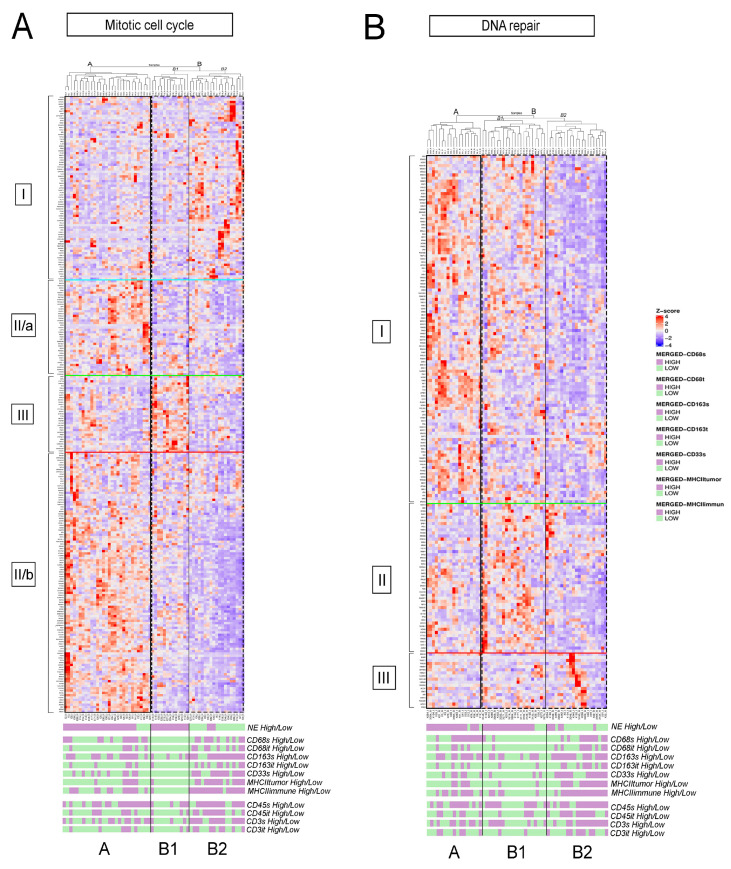
Gene expression analysis of oncogenic pathways. Heatmaps display gene expression profiles of individual samples (merged primary and LN) included in central oncogenic GO pathways. Every row represents a gene in the corresponding pathway, and every column represents a patient sample. Z-scores were determined according to the relative expression values of the HTG Edge oncological RNAseq panel. In the *mitotic cell cycle* pathway, cluster A tumors show strong expression of genes in cluster II and II/b. Cluster B1 is from exclusively NE-high, macrophage- and lymphocyte-low tumors (92% and 77%, respectively). These tumors overexpress genes in cluster III, whereas they underexpress all the other genes of the pathway. Cluster B2 is composed of mostly NE-low tumors (84%), subclassified into 70% macrophage-high and 66% lymphocyte-high. Interestingly, these tumors show decreased expression of all genes, except for those in cluster I (**A**). In the *DNA repair* GO pathway cluster A mostly represents NE-high (89%) and macrophage-low (61%) and lymphocyte-low (61%) tumors in the majority, and show strong expression of cluster I genes. Cluster B1 predominantly comprises NE-high tumors that are also macrophage- and lymphocyte-low (85% and 77%, respectively). These tumors show strong expression for cluster II genes and moderate expressions for genes of cluster I. Cluster B2 consists mostly of NE-low tumors exhibiting mainly macrophage- and lymphocyte-high phenotypes (63% and 62%, respectively). These tumors underexpress most genes in the pathway, except for cluster III (**B**).

Primary tumors and LN metastases showed similar pathway expression profiles (Figure 3); therefore, we pooled primary tumor and LN metastasis samples on pathway heatmaps. Figure 4 shows relative gene expressions of representative GO pathways generally upregulated in NE-high tumors and in tumors with low macrophage-related parameters (macrophage-low), but downregulated in NE-low tumors and tumors with high macrophage-related parameters (macrophage-high), including *mitotic cell cycle* (Figure 4A), and *DNA repair* (Figure 4B). We also used expression data from our earlier study regarding CD45 and CD3 expression in tumor and stroma [9], classifying tumors into high and low groups (Tables S2–S4). Tumors with increased CD3 expression were further labeled as “T-cell high”. NE-high tumors occur mostly in the first two clusters (A and B1, or A1 and A2) in oncogenic pathways, showing variable or weak association to all macrophage and T-cell densities. However, the last tumor clusters (B2 and B) consist mainly of NE-low, macrophage- and T-cell-high tumors (Figure 4B, Figure S6). Figure S4 shows the relative gene expressions of additional GO pathways, including *chromosome organization*, *mitotic sister chromatid segregation*, *DNA conformation change*, *and telomere organization* (details in figure legends).

**Figure 5:**
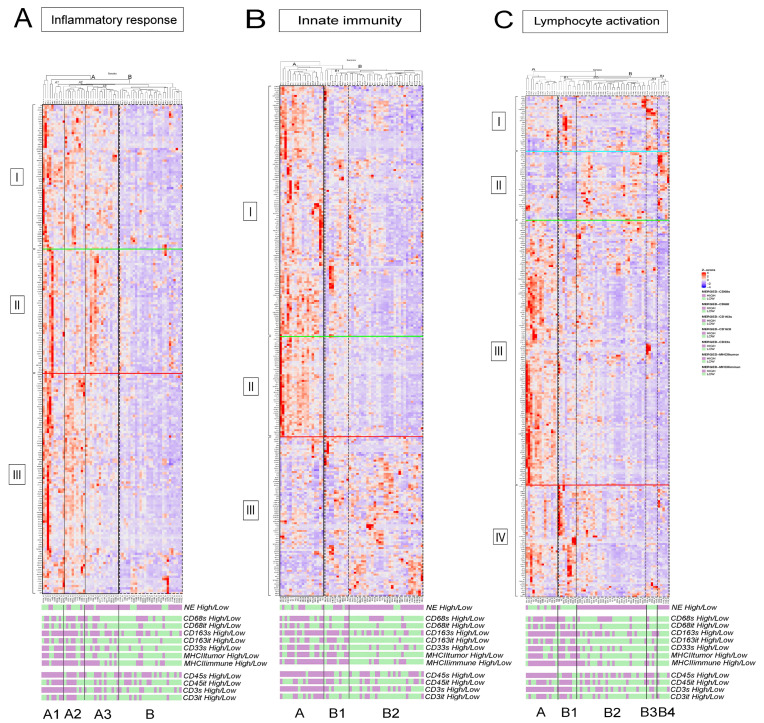
Gene expression analysis of immune response pathways. Heatmaps display gene expression profiles of individual samples (merged primary and LN) included in the most important GO pathways related to immune response. Every row represents a gene in the corresponding pathway, and every column represents a patient sample. Z-scores were determined according to the relative expression values of the HTG Edge oncological RNAseq panel. In the *inflammatory response* pathway, NE-low tumors exhibiting increased macrophage and T-cell infiltration occupy cluster A1 and A2 (80% and 67%), showing strong expression scores in all gene clusters. Cluster A3 represents 93% NE-high tumors that show variable associations with macrophage and T-cell densities. These tumors overexpress genes in cluster II, whereas they underexpress all the other genes in the pathway. Cluster B comprises primarily NE-high tumors (79%), with a generally macrophage-low, T-cell-low phenotype. These tumors show low expression of all genes in the pathway (**A**). In the *innate immunity* GO pathway, cluster A mostly represents NE-low tumors (67%) and is associated with high macrophage and T-cell densities, showing strong expression of cluster I and II genes. Cluster B1 comprises tumors more or less equally from every phenotype, expressing cluster I and III genes moderately, and underexpressing genes of cluster II. Tumors in cluster B2 consist of overwhelmingly NE-high tumors (90%) and associated with low macrophage and T-cell densities. Interestingly, these tumors overexpress cluster III genes, showing low expression for all other gene clusters (**B**). In the *lymphocyte activation* GO pathway, cluster A represents more NE-low tumors (64%) than NE-high tumors, and is associated with moderate or high immune cell densities. These tumors show strong expression in genes of cluster III and moderate expression of genes in cluster IV. Tumors in cluster B1 consist mainly of NE-low tumors (87%) and are associated with moderate or high immune cell densities. These tumors overexpress cluster I and IV genes, showing low expression for all other gene clusters. Cluster B2 consists exclusively of NE-high tumors, is associated with low macrophage and lymphocyte densities, and shows moderate expression in cluster II genes and low expression in all the other genes of the pathway. Cluster B3 includes a small subset of NE-low tumors, mostly associated with low macrophage and T-cell densities. These tumors show strong expression of genes in cluster I, and underexpress all the other genes in the pathway. Cluster B4 includes a small subset of NE-high tumors, mostly associated with low macrophage- and T-cell densities, showing strong expression of genes in cluster II, underexpressing all the other genes in the pathway (**C**).

Figure 5 shows the relative gene expressions of representative GO pathways upregulated in NE-low and immune-oasis tumors, but downregulated in NE-high and macrophage-low tumors, including GO pathways *inflammatory response* (Figure 5A), *innate immunity* (Figure 5B) and *lymphocyte activation* (Figure 5C). There are two main clusters (A and B) in the *inflammatory response* pathway.

Tumors in cluster A show strong expression in most inflammation-related genes, and can be divided into two subsets: NE-low subclusters (subcluster A1 and A2) and an NE-high cluster (subcluster A3). These tumors are associated with variable or high levels of macrophage- and T-cell densities. Cluster B represents NE-high tumors that possess macrophage-low and T-cell low phenotypes with decreased expression in every gene cluster (Figure 5A). The *innate immunity* pathway also shows strong upregulation in NE-low and strongly infiltrated tumors (Figure 5B). The NE-low, macrophage- and T-cell-high cluster A and the NE-high, macrophage- and T-cell-low cluster B show apparent distinction in their gene expression profile.

Interestingly, in the *inflammatory response* pathway, 28% of NE-low tumors (cluster B) exhibit an immune-desert phenotype, whereas 13% of NE-high tumors (in cluster A1 and A2) show strong expression of inflammatory response genes (Figure 5A). The same phenomena are observable for the *lymphocyte activation* pathway; a subset of NE-high tumors are associated with increased macrophage and lymphocyte densities and an immune-oasis transcriptomic phenotype. In contrast, a subset of NE-low tumors expresses a smaller, distinct set of genes and displays low expression for all the other gene clusters (Figure 5C, Figure S6). CSF1, a key factor in macrophage recruitment and survival, and VEGFC, an effector of angiogenesis and M2 macrophage-polarization show significantly higher expression in NE-low vs. NE-high tumors (7.23 ± 1.13 vs. 5.91 ± 1.05, *p* < 0.001 and 7.59 ± 1.46 vs. 5.95 ± 1.3, *p* < 0.008, Figure S6). Interleukin and chemokine receptors IL4R and CXCR2—pivotal molecules in alternative macrophage activation and immunosuppression—show significantly higher expression in the NE-low tumor phenotype compared to NE-high (8.71 ± 2.16 vs. 7.88± 1.84, *p* = 0.009 and 5.5 ± 1.02 vs. 3.83 ± 2.11, *p* < 0.001, Figure S6).

Figure S5 shows heatmaps of transcriptomic data of other pathways upregulated in NE-low tumors. In GO pathways, such as *regulation of cytokine production* and *NFKB signaling* NE-high tumors express a smaller and distinct set of genes compared to NE-low tumors. Genes for *extracellular structure organization* are prominently upregulated in NE-low and downregulated in NE-high tumors. A detailed analysis of tumor clusters and transcriptomic data is described in the figure legends of Figure 4 and Figure 5 and Figures S4 and S5.

### 3.4. Molecular Targets and Networks

**Figure 6:**
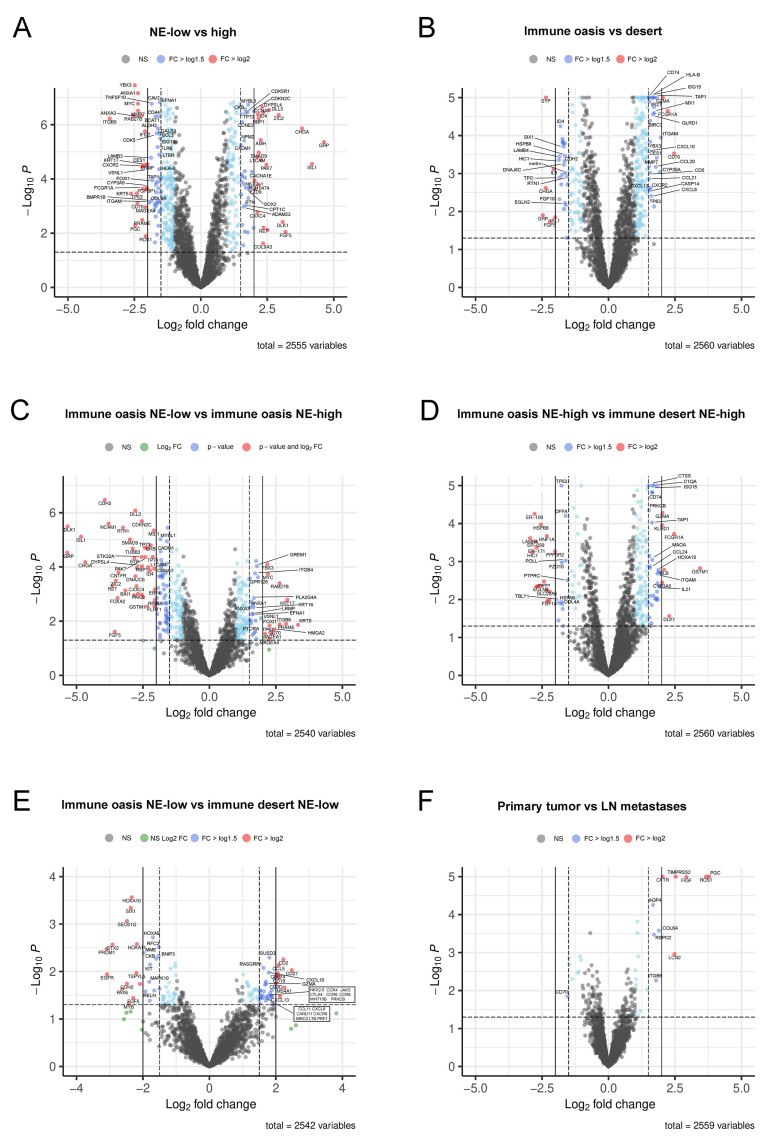
Molecular targets in different SCLC subtypes. Volcano plots show the comparison of gene expressions by NE phenotype and the extent of immune infiltration. Each dot represents a gene; the axis X displays the log2-transformed *p*-values from logistic regression, genes with *p* < 0.05 and Log2 FC value > 2 are colored red, genes with *p* < 0.05 and 1.5 < Log2 FC value < 2 are colored in dark blue and annotated with names. Dots representing genes with significant p-values, but Log2 FC values below 1.5 are colored in light blue. *p*-values were generated after the Bonferroni adjustment. Volcano plots show gene expression changes for NE-low (−Log2 FC) vs. NE-high tumors (+Log2 FC) and for immune-desert (−Log2 FC) vs. immune-oasis tumors (+Log2 FC) (**A**,**B**). Volcano plot compares NE-high immune-oasis (−Log2 FC) vs. NE-low immune-oasis tumors (+Log2 FC) (**C**), whereas a different plot compares immune desert NE-high (−Log2 FC) vs. immune oasis NE-high tumors (+Log2 FC) (**D**). Gene expression changes between immune desert NE-low (−Log2 FC) and immune oasis NE-low tumors (+Log2 FC) are shown in €. The alternative expression profile of primary tumors vs. LN metastases are shown in (**F**).

Next, we aimed to identify potential molecular targets showing increased or decreased expression in different tumor subsets. We pooled primary and LN samples, based on the molecular homogeneity demonstrated earlier in the pathway analyses (Figure 3, Figure 4 and Figure 5). According to biomarker high and low annotations (Figure 6), we analyzed NE-low vs. NE-high tumors (Figure 6A), and, separately, immune-desert and immune-oasis tumors (Figure 6B) in volcano plots.

To distinguish NE-specific molecular features within the immune-oasis phenotype, we compared NE-low immune-oasis vs. NE-high immune-oasis tumors (Figure 6C). Then, to assess the alternative expression profiles within a specific NE-phenotype, we juxtaposed immune-oasis vs. immune-desert NE-low and NE-high tumors, respectively (Figure 6D,E). A volcano plot comparing primary tumors to LN metastases is shown in Figure 6F.

**Figure 7:**
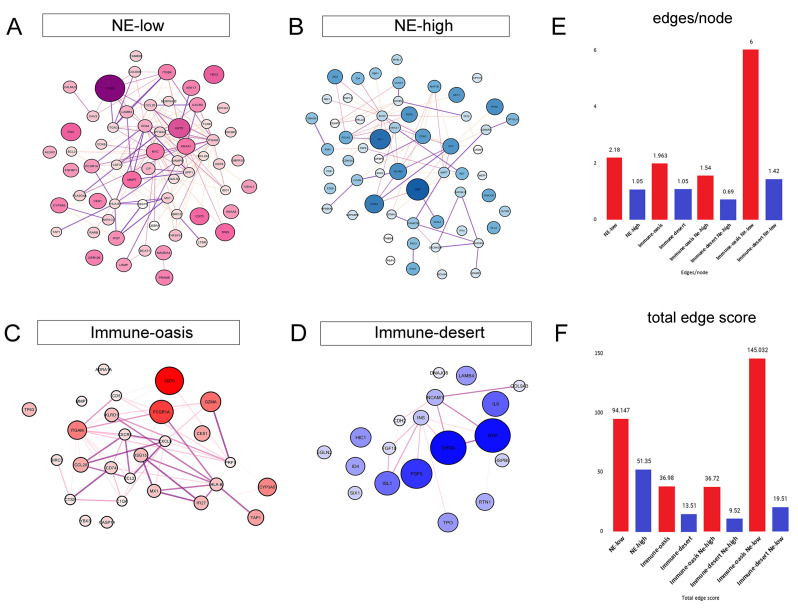
Network mapping of molecular targets. String maps represent the molecular network among differentially expressed genes in respective tumor phenotypes. Genes upregulated in NE-low tumors (vs. NE-high) show strong interconnectedness with an average of 2.18 edges/node and a 94.15 total connection score (**A**). Differentially expressed genes in NE-high tumors (vs. NE-low) show a moderate level of interconnectedness, with an average of 1.18 edges/node and a 51.35 total connection score (**B**). Genes upregulated in immune-oasis tumors (vs. immune-desert) show moderate interconnectedness with an average of 1.96 edges/node and a 36.98 total connection score (**C**). In immune-desert (vs. immune-oasis) tumors, upregulated genes represent weak interconnectedness, with an average of 1.05 edges/nodes and a 13.51 total connection score (**D**). Bar charts display the quantification of edges/node and total edge scores in different tumor phenotypes (**E**,**F**).

Next, we analyzed differentially expressed genes using string maps to visualize the interconnectedness and the network perspective (Figure 7A–D, Figure S10). We used the *p*-values and LogFC values derived from the previous analysis (Figure 6A–E). Generally, NE-low and immune-oasis tumors show a higher level of interconnectedness than NE-high and immune-desert tumors. Interestingly, when comparing immune-oasis NE-low with immune-oasis NE-high tumors (eliminating unambiguously immune-infiltration related genes from the analysis), nodes display a lower level of interconnectedness than NE-high tumors (Figure S10A,B). Comparing immune-oasis to immune-desert NE-high tumors reveals a higher level of interconnectedness in immune-oasis tumors (Figure S10C,D). Next, to analyze genes irrespective of NE-low-related genes, we found that immune-oasis NE-low tumors versus immune-desert NE-low tumors show more extensive interconnectedness of their genes by orders of magnitude (Figure S10E,F). Quantification of nodes and edges and interaction scores in every tumor subset is described in Table S5.

**Figure 8:**
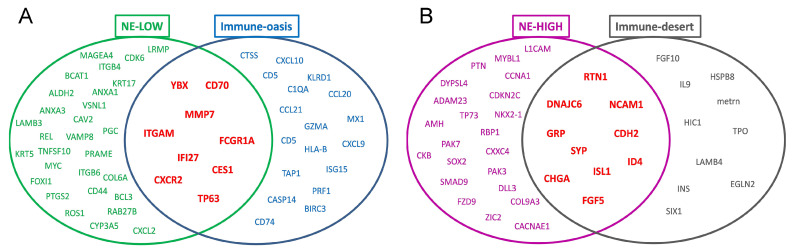
Summary of potential targets in NE-low vs. NE-high and immune-oasis vs. immune-desert SCLC. Venn diagrams display sets and section of genes upregulated in the overlapping groups of NE-low and immune oasis (**A**) and NE-high and immune-desert tumors (**B**), respectively.

Venn diagrams demonstrate the overlap of gene expression profiles between NE-low vs. immune-oasis and NE-high vs. immune-desert tumors (Figure 8), generated from the volcano plot analyses. NE-low tumors show specifically increased expression of ITGB6, ITGB4, KRT5, ANXA1, MYC, MMP7, CD44, ITGAM, HLA-B and CXCR2 vs. NE-high tumors, whereas immune-oasis tumors overexpress CD70, GZMA, FCGR1A, ITGAM, CXCR2, CXCL9, HLA-B and IFI27 vs. immune-desert tumors. NE-high tumors (vs. NE-low) mainly overexpress factors responsible for neural or neuroendocrine differentiation, including GRP, ISL1, CHGA, CDH2, FGF5, SYP, NCAM1, SOX3 and NKX2, whereas immune-desert tumors show increased expression for SYP, CHGA, FGF5, ISL1, INS, NCAM1 and CDH2, very similar to the NE-high gene network. Volcano plots and occurrence matrices of molecular sets and sections for the original parameters (CD68s, CD68t, CD163s, CD163t, CD33s, MHCII tumor, CD3s, and CD3t) are shown in Figures S7 and S8. Table S6 demonstrates molecular targets with possible therapeutic interventions, listing drugs already available and those under clinical trials.

**Figure 9:**
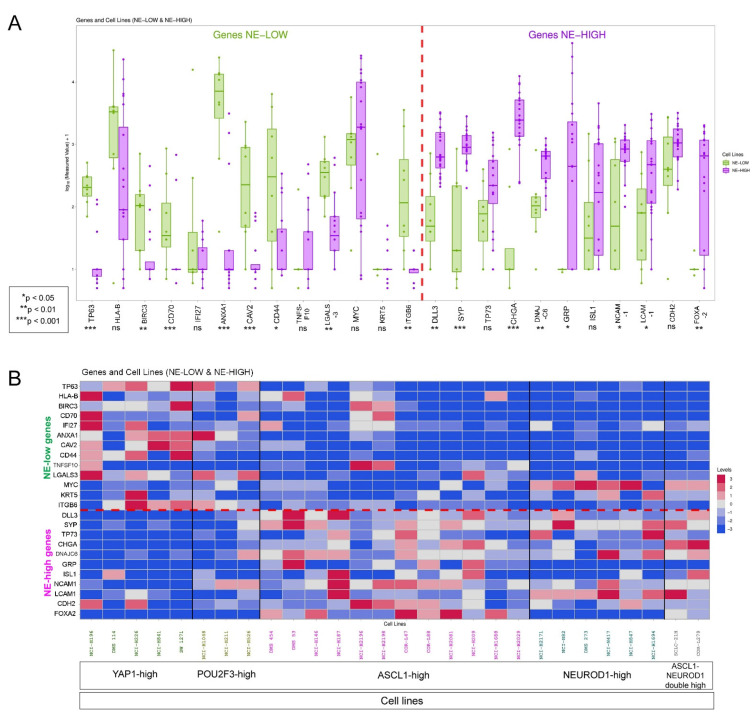
Cell line expression of key molecular targets. Plot chart and matrix show the expression of selected key molecular targets in NE-low and NE-high SCLC cell lines. Bar charts compare relative protein expression of key genes. Values are displayed on a logarithmic scale; the level of significance is displayed by asterisks. The Mann–Whitney test was used to compare means of expression data, and error bars indicate standard deviation (SD). In the case of NE-low targets, the difference was significant for TP63 (23.5 vs. 1.78, *p* < 0.001), BIRC3 (16.25 vs. 4.4, *p* < 0.01), CD70 (17.33 vs. 4.78, *p* < 0.001), ANXA1 (899.62 vs. 26.22, *p* < 0.001), CAV2 (61.87 vs. 1.45, *p* < 0.001), CD44 (156.56 vs. 4.54, *p* < 0.05), LGALS3 (43.75 vs. 9.4, *p* < 0.01) and ITGB6 (78.37 vs. 0.63, *p* < 0.01), but not significant for HLA-B (599.32 vs. 267.96, *p* = 0.14), IFI27 (200.3 vs. 1.4, *p* = 0.51), TNFSF10 (2.43 vs. 6.01, *p* = 0.23), MYC (148.75 vs. 607.16, *p* = 0.4) and KRT5 (9.1 vs. 0.87, *p* = 0.89) comparing NE-low vs. NE-high cell lines. Among NE-high targets, the difference was significant for DLL3 (16.37 vs. 109.65, *p* < 0.01), SYP (17.02 vs. 112.9, *p* < 0.001), CHGA (13.31 vs. 375.7, *p* < 0.001), DNAJC6 (18.12 vs. 59.1, *p* < 0.01), GRP (0.11 vs. 477.25, *p* < 0.05), NCAM1 (36.63 vs. 84.75, *p* < 0.05), L1CAM (18.8 vs. 81.95, *p* < 0.05) and FOXA2 (0.47 vs. 69.17, *p* < 0.01), but not significant for TP73 (12 vs. 42.8, *p* = 0.06), ISL1 (27.12 vs. 66.15, *p* = 0.32) and CDH2 (95.58 vs. 125.55, *p* = 0.19) comparing NE-low vs. NE-high cell lines (**A**). The matrix shows the relative protein expression values of key NE-low and NE-high genes in individual cell lines, further classified according to their NE-subtype marker expression as YAP1-high and POU2F3-high (NE-low) and ASCL1-high, NEUROD1-high and ASCL1-NEUROD1 double high (NE-high) (**B**). Individual scaling was used for molecular targets in every row.

To validate our transcriptomic data on key NE-high vs. NE-low molecular targets, we cross checked their protein expression on representative SCLC cell lines classified as NE-high and NE-low according to NE-subtype protein expression. Figure 9A shows the comparison of relative protein expression values of key molecular targets in NE-low vs. NE-high cell lines, and Figure 9B demonstrates the protein expression level for individual cell lines in a color-coded matrix.

## 4. Discussion

SCLC is a highly progressive cancer with an unmet need for therapeutic advancements. Crosstalk between tumor cells and other tumor-associated cells in the TME is crucial in tumor progression, and paves the way for SCLC drug developments. Tumor-infiltrating immune cells, such as TAMs, lymphocytes, or dendritic cells, are endowed with a double-edged sword characteristic [36,37], and the heterogeneity and function of TAM phenotypes can have multiple ramifications, in the TME in particular. Recruitment to the tumor site and exposure to TME-derived factors (cytokines, chemokines, growth factors, reactive oxygen species, or hypoxia) can polarize TAMs from tumoricidal to tumorigenic. Eventually, the loss of TAMs’ cytotoxic ability and their capability to produce pro-inflammatory cytokines may establish substantial barriers to solid tumors’ immune clearance [38].

In this study, our novel findings include the fact that TAMs are the most populous cell type of the TME in limited-stage SCLC, especially in tumor nests, where their cellular density far exceeds the number of T-cells. Another crucial finding shows that most CD68 + TAMs are M2-polarised, and express CD163 in NE-low tumor subsets, creating an immunosuppressive microenvironment peculiarly inside tumor nests. Primary tumors and matched LN metastases show similar cellular infiltrations; however, CD3 + T-cells occur more frequently, whereas CD163 + M2-TAMs are present in lower numbers in LN metastases. This is in line with ovarian carcinoma [39], but in contrast with breast cancer studies [40], showing distinct immune mechanisms across tumor types. In SCLC, the number of CD33+ myeloid cells is relatively low, mainly present in the stroma of NE-low tumors with a high ratio of M2-polarised TAMs. This contrasts with a report of a subset of NSCLC tumors with extensive CD33+ myeloid cell infiltration [30], meaning that this cell population might play a different role in SCLC TME.

The expression of the MHCII molecule in cancer cells in the vicinity of tumor-infiltrating immune cells was reported earlier in PDXs [41] and SCLC [9]. Here, we show that strong MHCII expression in tumor cells is coincident specifically with CD68- and CD163-high subsets of NE-low tumors. Tumor-specific MHCII expression may increase tumor recognition by the immune system and has been associated with an increased number of TILs, superior tumor rejection in rodents, and better prognosis in clinical practice [42,43,44]. The massive co-occurrence of TAMs and M2-polarised macrophages in these tumors can diminish the anti-tumor effects of MHCII expression. Interestingly, a high number of CD3 + TILs is associated with high CD68 and CD163 expression in tumor nests, but not in the stroma, implicating a distinct colonization mechanism for lymphocytes and macrophages in various tumor compartments. Nonetheless, the presence of generally tumoricidal TILs in tumor nests, subsequently followed by the colonization of TAMs, may suggest an immune evasion mechanism for tumor cells.

Next, we performed an fGSEA analysis according to the GO pathway iteration. We found extensive overlap in pathway expression profiles between NE-low and immune-oasis tumors, irrespective of tumor localization (primary tumor or LN metastasis). These tumors both exhibited increased, positive enrichment scores in immunological activity pathways—remarkably those associated with inflammatory response or innate immunity—compared to NE-high and immune-desert tumors. In contrast, oncogenic pathways associated with cell proliferation and DNA-repair were downregulated in the NE-low and immune-oasis group and upregulated in NE-high and immune-desert tumors, disregarding primary or metastatic tumor site. Interestingly, we identified a subset of NE-high tumors representing an immune-oasis phenotype with strong upregulation of inflammatory factors. In contrast, we found that a limited subgroup of NE-low tumors is immune-deserted and express distinct cellular pathways. Despite the fact that LN metastases showed minor differences in immune cell infiltration, including increased T-cell densities and a decreased number of M2-TAMs, their molecular expression profile in terms of immune response exhibits striking similarity with their matched primary tumor.

Afterward, we implemented the immune-oasis/-desert distinction and narrowed it down to particular NE phenotypes. Using volcano plots and string maps to visualize their connectome, we revealed potential new molecular targets. NE-low tumors exhibit a striking overlap in gene expression profile with immune-oasis tumors; however, specific genes showed an association with NE-low rather than immune-oasis tumors. Annexin A1 (ANXA1) is strongly upregulated in NE-low tumors irrespective of immune infiltration, promoting immunosuppression. Its molecular mechanism is similar to glucocorticoids, promoting TGFB-signaling, resolving inflammation, and wound-healing [45]. Others showed that ANXA1 is upregulated in melanoma [46], hepatocellular carcinoma [47], gastric cancer [48] and lung adenocarcinoma [49], which correlates with poor prognosis. Furthermore, overexpression of ANXA1 was reported to increase resistance to chemoradiotherapy in colorectal [50] and breast cancer [51]. Strong evidence underlines the role of ANXA1 in epithelial-mesenchymal transition (EMT) and alternative macrophage polarization, pivotal in tumor invasion and metastasis [52,53]. This is in line with our gene expression analysis, where CD163-high tumors showed increased expression of ANXA1 compared to CD163-low tumors (Figure S6). ShRNA-mediated silencing of ANXA1 suppresses nuclear factor (NF)-kB activity, which results in the direct inhibition of CXCR4 and matrix metalloproteinase (MMP) expression, impeding tumor cell invasion [54].

Other promising molecular targets we identified are CD70 and its receptor CD27, upregulated in both NE-low and immune-oasis tumors. Physiologically, TNF receptor superfamily member, CD27, plays a costimulatory role in T-cell expansion through the NFκB pathway [55]. Its ligand, CD70, is only transiently expressed on activated T-cells, B-cells, and mature dendritic cells [56], while its overexpression has been reported in renal cell carcinoma [57] and hematological malignancies [58]. Moreover, it was demonstrated that CD70 plays a crucial role in evoking immune surveillance by recruiting CD27+ regulatory T-cells (Treg) to the tumor site [59]. The cytokines’ expression was recently shown in NSCLC cell lines and FFPE samples [60].

The involvement of cytokines, chemokines, and their receptors in tumor growth and progression was demonstrated by multiple studies, showing that they can stimulate tumor cell survival and proliferation in many ways. Th1-biased immune response was proved to have utmost importance in efficient anti-tumor reaction and mediated by T-cells and M1-polarised macrophages [61]. Chemokine receptor CXCR3 and its ligands CXCL9 and CXCL10 [62] are all inherent in Th1-biased immune responses, including in the recruitment of tumoricidal immune cells [63], subsequently hallmarking the role of the central pro-inflammatory factors in immune-oasis SCLC according to our data (Figure 7). Another tumoricidal factor, granzyme A, is secreted by cytotoxic T-cells mediating direct tumor cell lysis [64]. In our study, it is significantly overexpressed in immune-oasis, but not in NE-low tumors. On the contrary, several sets of chemokines and their receptors, such as CCR2/CCL2, CXCR2/CXCL5, and VEGF, have been reported to promote tumor progression in other tumors by enhancing immunosuppressive M2-TAM and MDSC function [65]. According to our expression data, CXCR2 seems to fulfill a crucial role in establishing an immunosuppressive TME in NE-low tumors, irrespective of immune infiltration. MDSCs acquire their immunosuppressive properties in the tumor microenvironment (TME) through the NFκB signalization pathway [66]. According to our transcriptomic data, the NFκB pathway shows significant overexpression, including the BIRC3, REL, IFI27, and TNFSF10 genes in NE-low CD163-high tumors, harboring the highest infiltration of CD33+ myeloid cells.

Next, we analyzed genes associated with extracellular structure organization (MMP7, ITGB4, ITGB6, LAMB3, COL6A, and LGALS3) that were significantly more expressed in NE-low tumors. MMP7 overexpression in NE-low tumors is not explicable only by the mere increase in the number of infiltrating immune cells (characteristic in the NE-low phenotype) because it is not upregulated in immune-oasis NE-high tumors (Figure S10). Furthermore, it was reported that increased MMP7-secretion was associated with tumor proliferation, decreased response to chemotherapy, and a poor prognosis in NSCLCs [67]. This may suggest a more invasive cancer cell phenotype in NE-low tumors. Integrin alpha-V beta-6 (ITGB6) is a receptor for fibronectin and cytotoxin, and its internalization via clathrin-mediated endocytosis promotes carcinoma cell invasion [68]. Moreover, ITGB6 mediates R-G-D-dependent release TGFβ, thereby playing a pivotal role in activating the immunosuppressive TGFβ pathway [69].

Another selectively NE-low phenotype-related molecule, RNA-binding, cold-shock protein YBX3 seems to act as a translational repressor binding the promoter of CSF3 [70]. Its function and oncological implications are not yet clarified in lung cancer, making it a suitable target for further research. Keratin 5 (KRT5), a member of the microfibrillar keratin type II family, which serves as a biomarker for several types of cancer, including lung cancer [71] and triple-negative breast cancer [72] was found to be specific to the NE-low tumor subset. Bioinformatic analysis of RNAseq data also revealed that the anti-apoptotic oncogene, cyclin-dependent kinase 6 (CDK6) is overexpressed explicitly in NE-low compared to NE-high tumors, disregarding the immune infiltration pattern. CDK6 overexpression was categorically associated with a worse prognosis in NSCLC [73]. In contrast, TP63, another cell-cycle-associated protein, similarly overexpressed in NE-low tumors, was reported to be a tumor suppressor associated with a better prognosis in lung cancer [74].

According to a recent study, MYC can act as a master regulator for NE-differentiation and can dynamically shift tumor phenotype acting on the NOTCH signaling pathway from ASCL1 + NEUROD1+ (NE-high) to YAP1+ or POU2F3+ (NE-low) [75]. This innovative new approach is supported by our findings that MYC RNA-expression is increased in NE-low tumors (Figure 6A and Figure 7A) and by another study, claiming that MYC drives the progression of SCLC to variant NE-subtype [76], which was later reinterpreted as NE-low by Rudin and colleagues [5].

In our study, high-affinity IgG receptor 1A (FCGR1A) is significantly overexpressed in NE-low tumors (vs. NE-high) and in immune-oasis tumors (vs. immune-desert). FCGR1A is a novel finding, not reported previously in cancer, and may well play a role in mAb-based immunotherapy, based on the receptors’ potent cytotoxic effects and their capability to efficiently mediate antigen presentation [77]. The importance of antigen presentation in immune-oasis SCLC is further supported by MHCII-related molecules, such as HLA-B, TAP1, and CD74, which are all extensively overexpressed in this tumor phenotype (Figure 6) and strongly interconnected according to string maps and network analysis (Figure 7).

Interestingly, the molecular networks interpreted by string maps related to the immune response showed that it is more interconnected in NE-low immune-oasis tumors than in NE-high immune-oasis tumors. A higher level of connectivity among molecules means that signaling pathways depend more on each other. Therefore, targeting these central molecules might reach more extensive tumor inhibition. We also disclosed that NE-low and immune-oasis tumors exhibit significant overlap; still, a considerable number of NE-high tumors display increased immune infiltration, and a definitive subset of NE-low tumors are immune-desert. These alternative tumor subsets exhibit distinct gene expression patterns in critical immune response and oncogenic pathways that might justify different therapeutic approaches to these hybrid tumor phenotypes.

This study has limitations. First, it is a retrospective cross-sectional study with insubstantial outcome data available. However, disease aggressiveness might hamper the prognostic impact, especially in this limited-stage SCLC cohort without specific therapy administered. Despite the transcriptomic and cell line verification of our expression data, it is important to highlight that in situ proteomic validation of these targets is still required for the initiation of preclinical drug studies. Furthermore, our results concern only limited-stage SCLC, whereas advanced-stage disease might exhibit a different molecular behavior. The patient cohort is limited in size in terms of the number of patients included and the size of each sample in the TMA. Studies with higher case numbers are needed to comprehensively characterize SCLC tumor subsets with distinct gene expression profiles.

## 5. Conclusions

The therapeutic strategy in SCLC clinical guidelines is currently based on cancer cell-related attributes with no biomarker used in the clinical practice; the TME is yet not implemented in drug developments to stratify patients for therapy [12]. This study demonstrated that TAMs and many factors in the TME play a crucial role in limited-stage SCLC tumor biology. We believe that interpreting novel potential targets in the context of distinct, NE-associated TME patterns is the critical step forward for planning future research. Accordingly, we hypothesize that, despite increased immune infiltrations, NE-low tumors have a microenvironment associated with increased M2-TAM densities and expression of potentially immunosuppressive molecules, such as ANXA1, CXCR2, CD70 and ITGB6, and might benefit from future targeted immunotherapies. However, further studies in larger datasets are needed to confirm our findings. We recommend considering new drug developmental strategies and further validation based on these novel immune-related mechanisms to enhance new therapeutic approaches in this deadly cancer.

## Data Availability

Processed raw data for the HTG Edge Oncological RNAseq panel and sequencing data broken down to individual GO pathways (Z-scores for patients and genes listed in Figure 4 and Figure 5) is publicly available in the “Mendeley data” online repository: under the CC BY 4.0 license. Dataset tiltle: Dora, David (2021), “SCLC HTGEdge RNAseq dataset”, Mendeley Data, V1, doi:10.17632/7n6rk5zgjj.1.

## Supplementary Materials

The following are available online at https://www.mdpi.com/article/10.3390/biology10060502/s1, Figure S1: Regression analysis of macrophage (CD68, CD163, CD33), MHCII, immune cell (CD45), and lymphocyte (CD3) marker tissue expression in SCLC tumor samples, Figure S2: Distribution of CD68-, CD163-, CD33-, and MHCII HIGH- and LOW primary tumors and LN metastases according to NE phenotype, Figure S3: TOP20 up-and downregulated pathways in the fGSE analysis, Figure S4: Gene expression data of additional oncogenic pathways, Figure S5: Gene expression data of pathways on cytokine production, signaling, and extracellular structure organization, Figure S6: Composition of patient tumor clusters according to NE-phenotype, macrophage- and T-cell infiltration, Figure S7: Volcano plots for parameters of selected IHC biomarkers in different tumor compartments, Figure S8: Occurrence matrices for parameters of selected IHC biomarkers in different tumor compartments, Figure S9: Immune-oasis and immune-desert tumors and their NE-phenotype, Figure S10: Additional string maps for the comparison of SCLC tumor phenotypes, Table S1: Correlation between the tissue expression of different macrophage markers (CD68, CD163, and CD33), MHCII ex-pression on tumor cells, CD45 immune cell marker and CD3 T-lymphocyte marker in stroma and tumor nests, Table S2: Cut-off values for macrophage related IHC biomarkers (HIGH- and LOW tumor categories), Table S3: Cut-off values for IHC biomarkers CD45 and CD3 (HIGH- and LOW tumor categories), Table S4: HIGH and LOW annotation for NE-high and NE-low tumors according to protein marker expression, Table S5: Node and edge data for string maps, Table S6: Possible therapeutic interventions on eligible molecular targets.
