# Supplementary material for: Characterization of Tumor-Associated Macrophages and the Immune Microenvironment in Limited-Stage Neuroendocrine-High and -Low Small Cell Lung Cancer
Source: Biology (Basel). 2021 Jun 4;10(6):502. doi: 10.3390/biology10060502 (PMC8228874; doi:10.3390/biology10060502)
Supplement: Supplementary file 1 [file biology-10-00502-s001.zip › biology-1218389-Supplementary material.pdf]

SUPPLEMENTARY MATERIAL

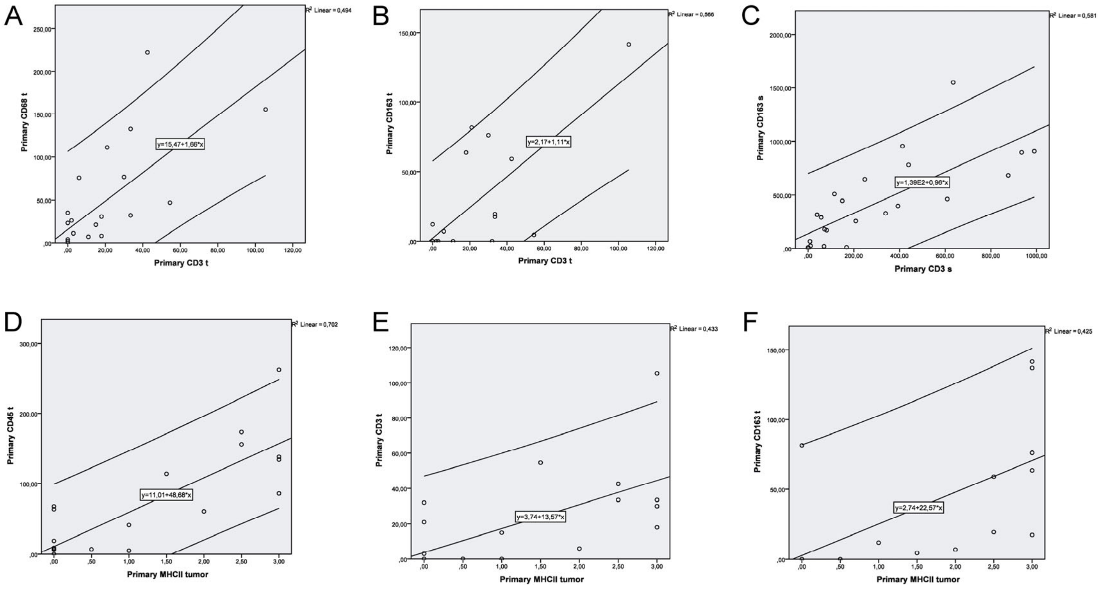

Figure S1. Regression analysis of macrophage (CD68, CD163, CD33), MHCII, immune cell (CD45), and lymphocyte (CD3) marker tissue expression in SCLC tumor samples.

The expression of CD68 and CD163 showed a very strong and strong positive correlation with CD3 expression in tumor nests (r=0.807, p<0.001; A and r=0.707, p<0.001; B), respectively. The cellular density of CD163+ cells showed very strong positive correlation with CD3+ T-lymphocyte density in the stroma compartment (r=0.864, p<0.001; C).

The cellular density of CD45+ immune cells and CD3+ T-lymphocytes in tumor nests showed a strong positive correlation with MHCII expression on tumor cells (r=0.753, p<0.001, D and r=0.705, p=0.001, E). Cellular density of CD163+ macrophages in tumor nests showed very strong positive correlation with MHCII expression on tumor cells (r=0.801, p<0.001, F).

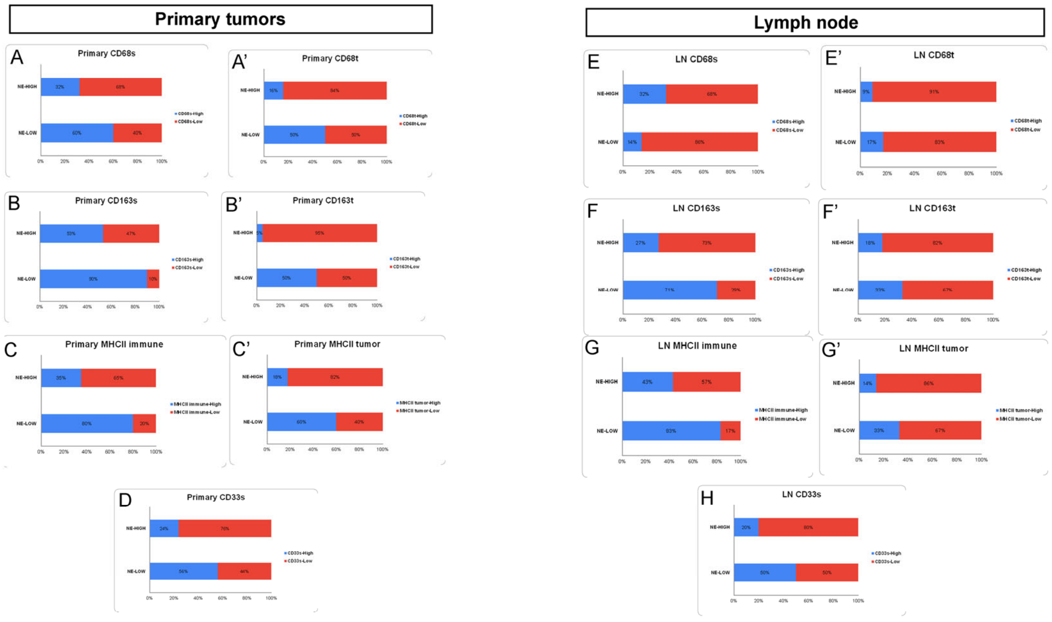

Figure S2. Distribution of CD68-, CD163-, CD33-, and MHCII HIGH- and LOW primary tumors and LN metastases according to NE phenotype.

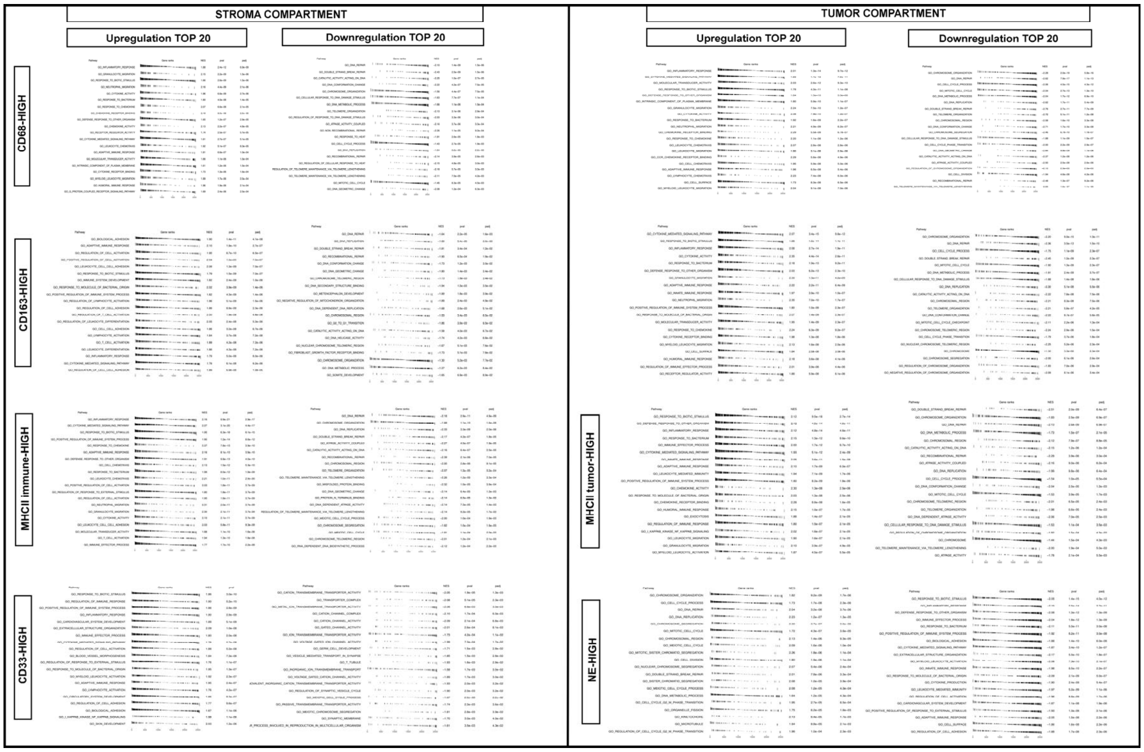

Figure S3. TOP20 up-and downregulated pathways in the fGSE analysis *NES: Normalised enrichment score; p-val: p-value; p-adj: adjusted p-value*

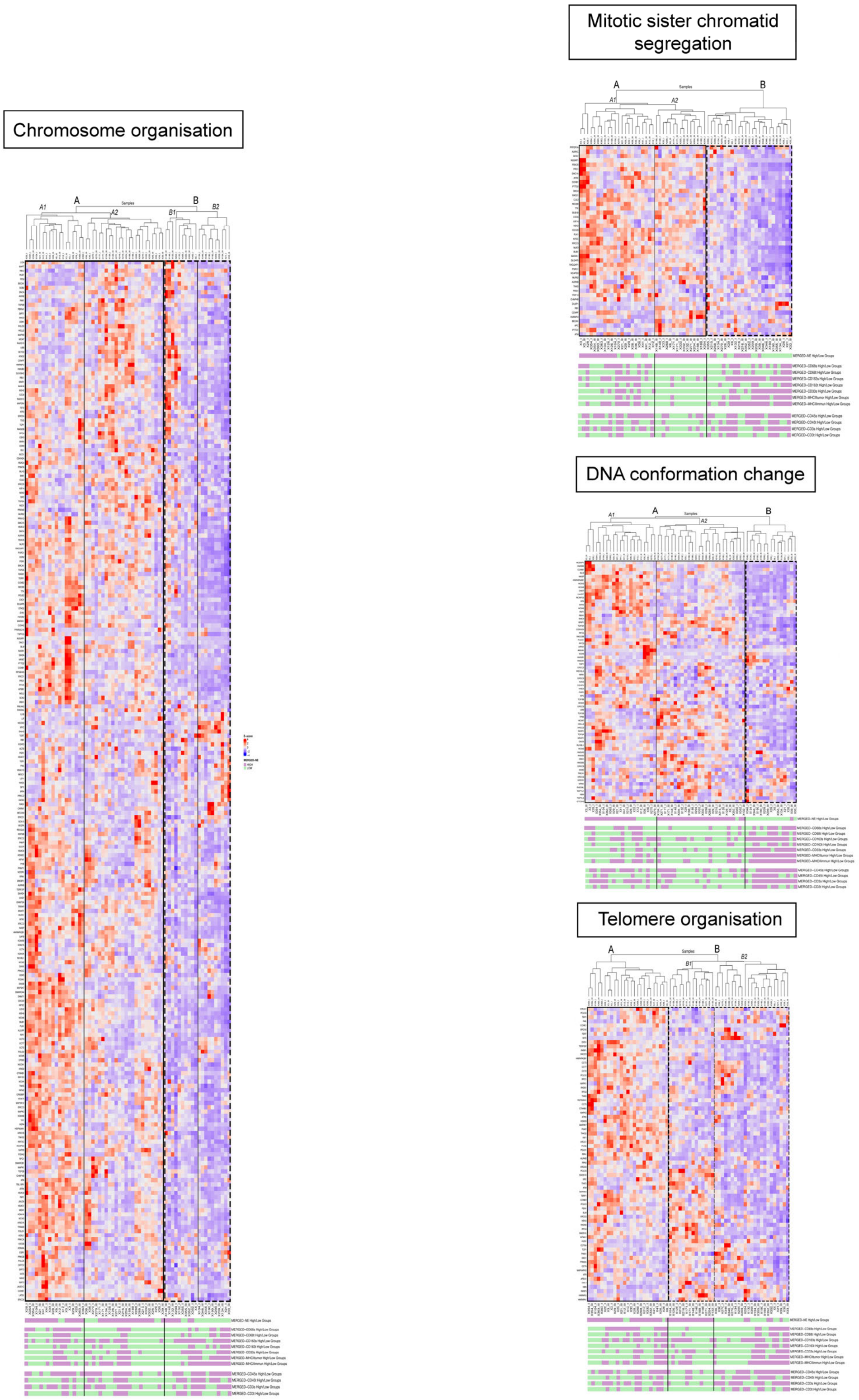

Figure S4. Gene expression data of additional oncogenic pathways

Heatmaps show gene expression profile of GO pathways, *chromosome organization*, *mitotic sister chromatid segregation*, *DNA conformation change,* and *telomere organization* at the level of individual tumor samples (primary and LN merged). The first two clusters (A1 and A2, A and B1 in *telomere organization*) represent mainly NE-high tumors with low or variable levels of macrophage- and T-cell infiltration. These tumors show strong overall expression in most genes of

their corresponding pathway. The third cluster (B, B2 in telomere organization) comprises NE-low phenotype tumors in all of the oncogenic pathways, where it is associated with macrophage-high and lymphocyte-high tumors.

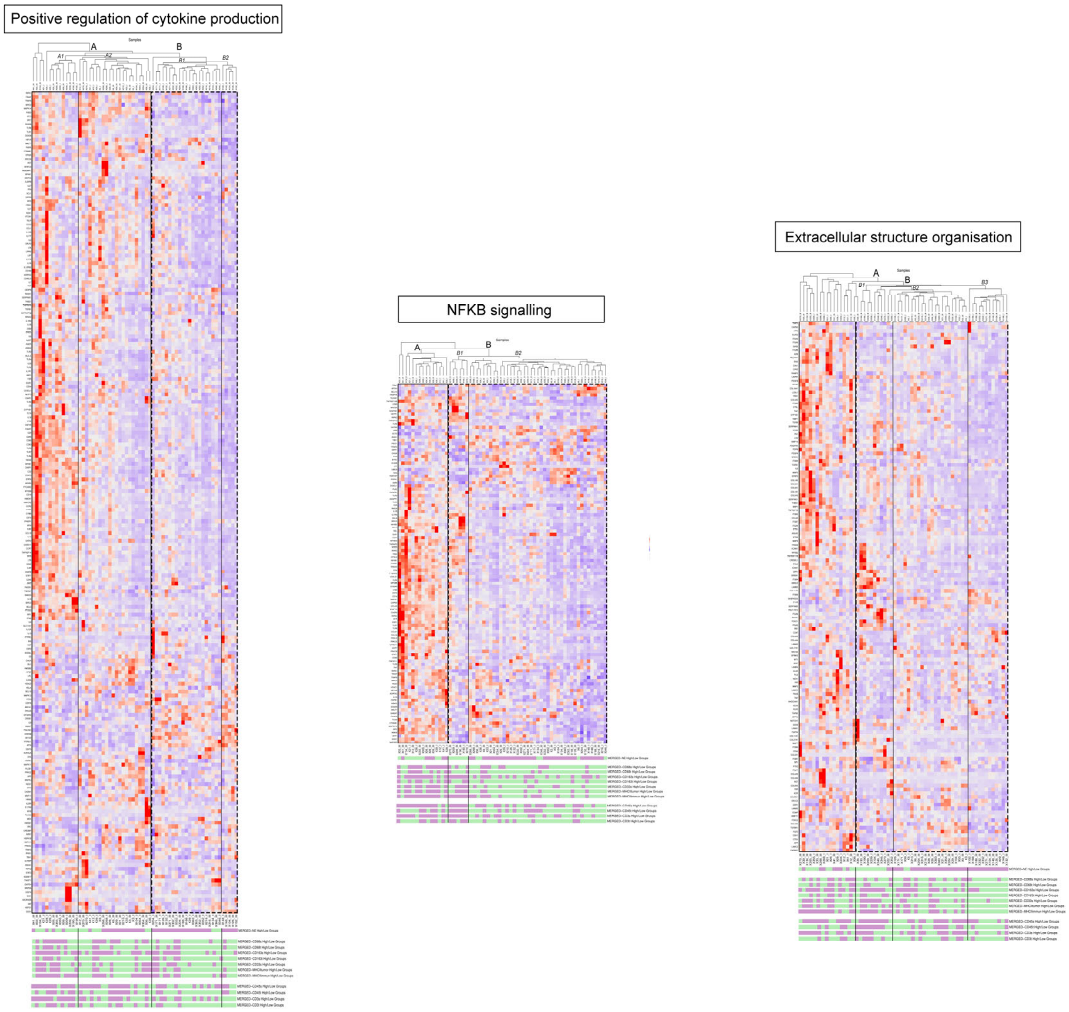

Figure S5. Gene expression data of pathways on cytokine production, signaling, and extracellular structure organization

Heatmaps show gene expression profile of GO pathways, *positive regulation of cytokine production*, *NFKB signaling* and *extracellular structure organization* at the level of individual tumor samples (primary and LN merged). The first two clusters (A1 and A2, A and B1 in *extracellular structure organization*) represent in majority NE-low tumors variable or high levels of macrophage- and T-cell infiltration. These tumors show strong overall expression in most genes of their corresponding pathway. The last clusters (B1 and B2 in *positive regulation of cytokine production,* B in *NFKB signaling,* and B2 and B3 in *extracellular structure organization*) comprises tumors of mainly NE-high phenotype, where they are associated with variable or low macrophage- and lymphocyte-densities.

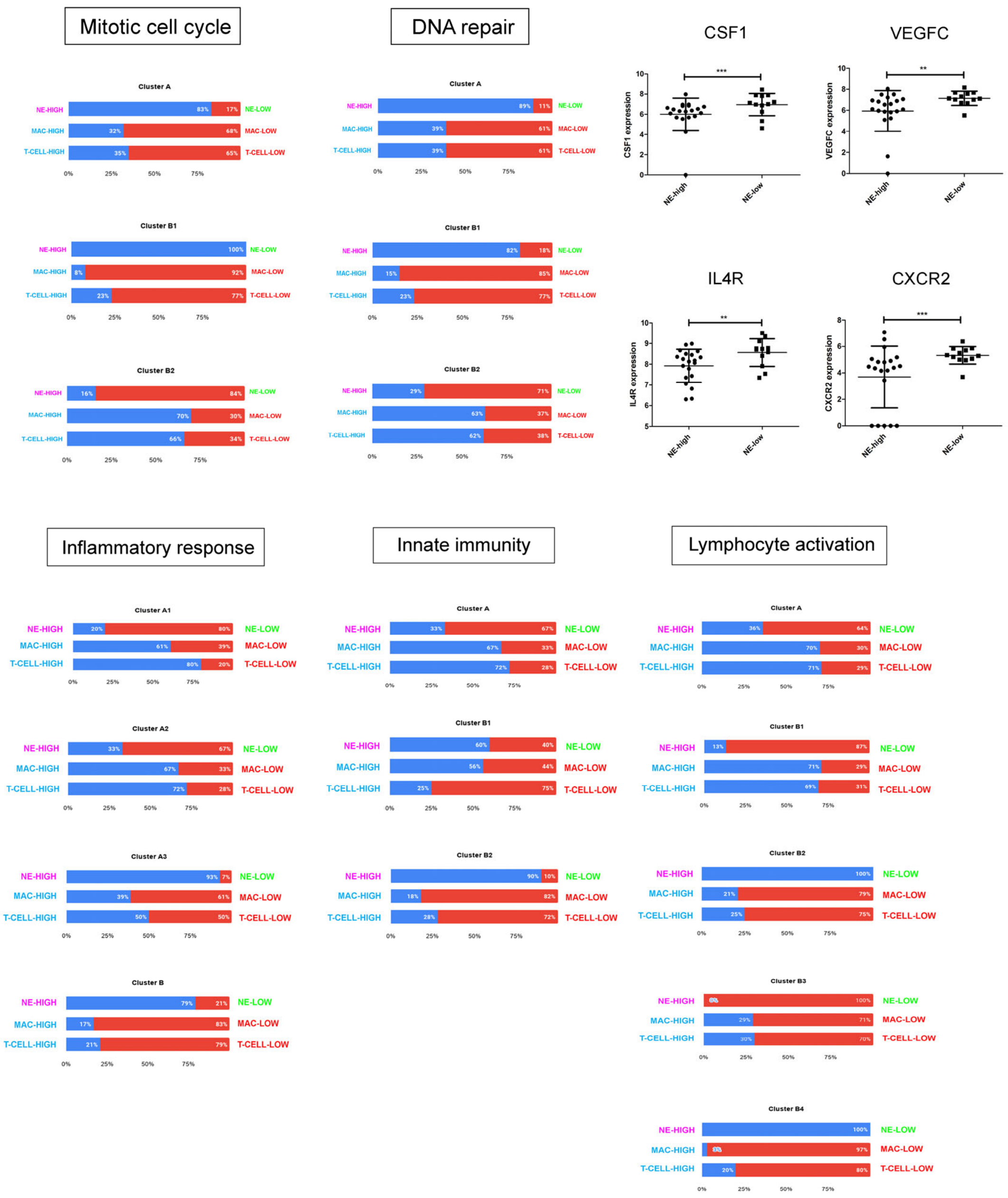

Figure S6. Composition of patient tumor clusters according to NE-phenotype, macrophage- and T-cell infiltration. Stacked bar charts show tumor-phenotype composition of different clusters according to oncogenic (*mitotic cell cycle, DNA-repai*r), and immunogenic (*inflammatory response, innate immunity, lymphocyte activation*) GO pathways. Scatter plots show the differential expression of CSF1, VEGFC, IL4R and CXCR2 in NE-low compared to NE-high tumors.

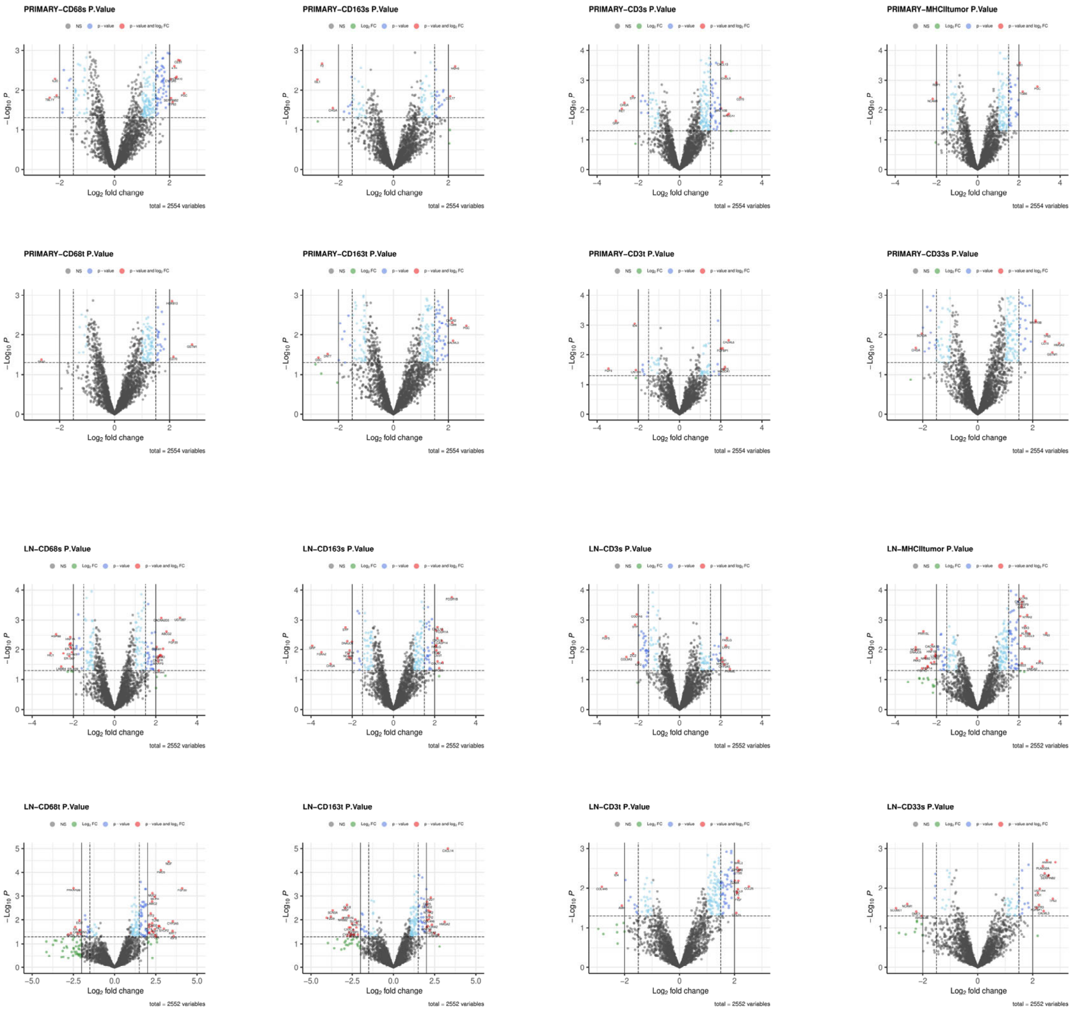

Figure S7. Volcano plots for parameters of selected IHC biomarkers in different tumor compartments

A

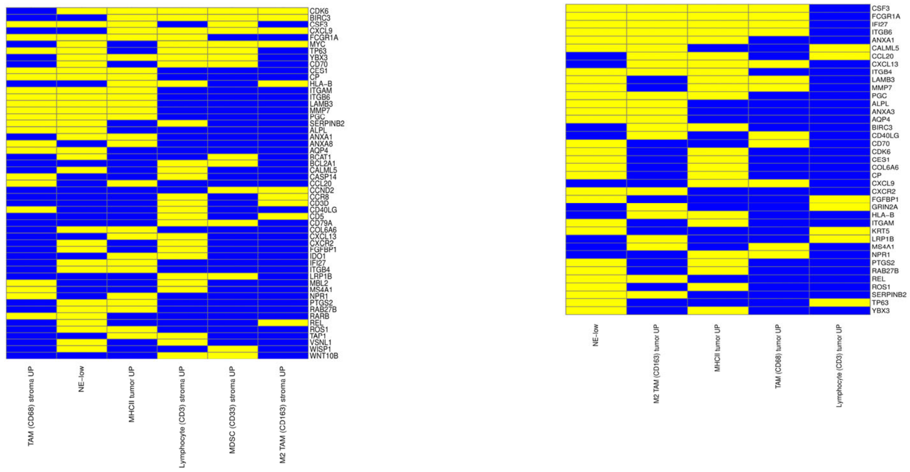

B

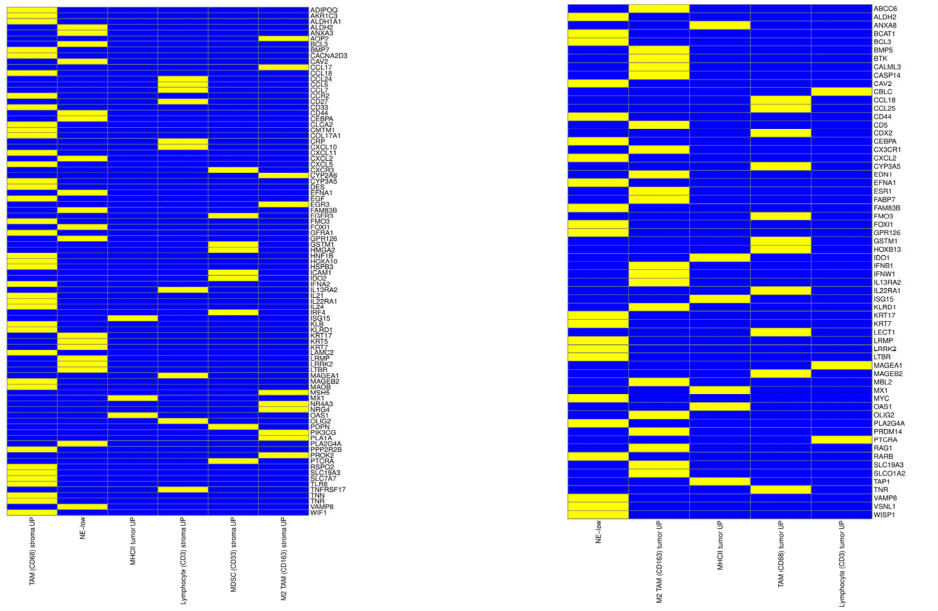

Figure S8. Occurrence matrices for parameters of selected IHC biomarkers in different tumor compartments
The occurrence of key genes in sets and sections of different parameters are shown in matrices for stromal (A) and intratumoral expressions (B). Only genes with p-value < 0.05 and LogFC value > 1.5 are included in the analysis.

Immun oasis tumors

NE-low	6-I
NE-low	21-I
NE-low	39-I
NE-low	270-I
NE-low	209-I
NE-low	190-I
NE-low	134-I
NE-low	12-III
NE-low	270-III
NE-low	209-III
NE-low	190-III
NE-low	33-III
NE-low	36-III
NE-low	134-III
NE-high	28-I
NE-high	33-I
NE-high	352-I
NE-high	3-I
NE-high	41-I
NE-high	171-III
NE-high	26-III
NE-high	28-III
NE-high	6-III
NE-high	29-III
NE-high	39-III

NE-low	NE-high
14	11

Immun desert tumors

NE-low	12-I
NE-low	346-I
NE-low	36-I
NE-low	346-III
NE-high	104-I
NE-high	126-I
NE-high	132-I
NE-high	148-I
NE-high	171-I
NE-high	188-I
NE-high	204-I
NE-high	210-I
NE-high	216-I
NE-high	268-I
NE-high	271-I
NE-high	29-I
NE-high	354-I
NE-high	363-I
NE-high	104-III
NE-high	126-III
NE-high	132-III
NE-high	148-III
NE-high	188-III
NE-high	204-III
NE-high	210-III
NE-high	216-III
NE-high	268-III
NE-high	21-III
NE-high	271-III
NE-high	352-III
NE-high	354-III
NE-high	363-III

NE-low	NE-high
4	28

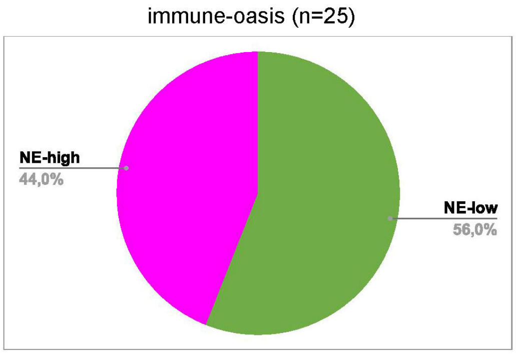

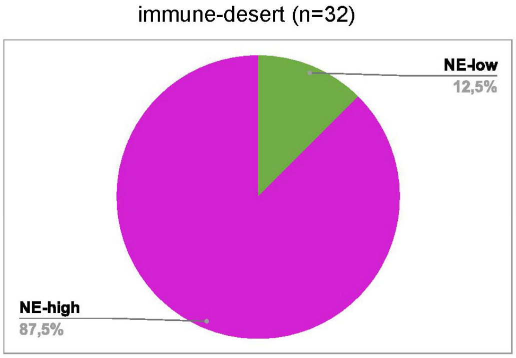

Figure S9. Immune-oasis and immune-desert tumors and their NE-phenotype

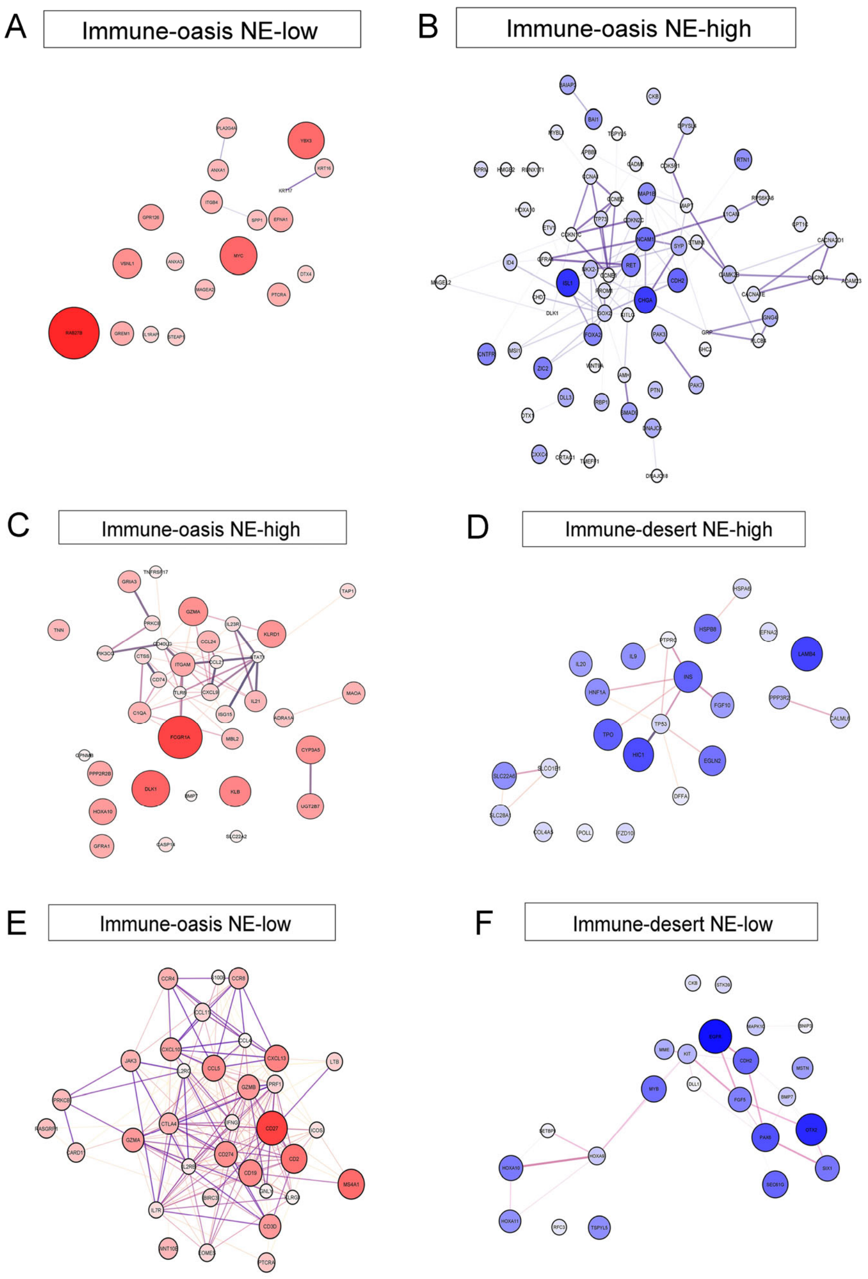

Figure S10. Additional string maps for the comparison of SCLC tumor phenotypes

In immune oasis NE-low tumors (vs immune oasis NE-high), the low number of differentially expressed genes show minimal interconnectedness, whereas central genes of immune oasis NE-high (vs immune oasis NE-low) tumors are interconnected moderately (A-B). Immune oasis NE-high tumors (vs immune desert NE-high) show a moderate level of interconnectedness, with an average of 1.54 edges/node and a total 36.72 connection score. Immune desert NE-high tumors (vs immune oasis NE-high) however represent weak interconnectedness with average 0.69 edges/node and 9.52 total connection score (C-D). Immune oasis NE-low (vs immune desert NE-low) tumors display strong interconnectedness among their differentially expressed genes with average 6 edges/node and 145.03 total connection score. In contrast, top genes of immune desert NE-low (vs immune oasis NE-low) tumors are interconnected weakly with average 1.42 edges/node and 19.51 total connection score (E-F).

Table S1. Correlation between the tissue expression of different macrophage markers (CD68, CD163, and CD33), MHCII expression on tumor cells, CD45 immune cell marker and CD3 T-lymphocyte marker in stroma and tumor nests.

MACROPHAGE MARKERS

PRIMARY	CD68s	CD68t	CD163s	CD163t	CD33s
CD68t	r=0.845 p<0.001				
CD163s	r=0.455 p=0.02	r=0.653 p=0.001			
CD163t	r=0.628 p=0.01	r=0.764 p<0.001	r=0.594 p=0.003		
CD33s	r=0.406 p=0.054	r=0.500 p=0.018	r=0.651 p=0.001	r=0.605 p=0.003	
MHCII tumor	r=0.570 p=0.006	r=0.703 p<0.001	r=0.511 p=0.018	r=0.801 p<0.001	r=0.505 p=0.019

LN	CD68s	CD68t	CD163s	CD163t	CD33s
CD68t	r=0.894 p<0.001				
CD163s	r=0.651 p<0.001	r=0.478 p=0.016			
CD163t	r=0.488 p=0.013	r=0.410 p=0.042	r=0.792 p<0.001		
CD33s	r=0.302 p=0.161	r=0.270 p=0.224	r=0.368 p=0.084	r=0.362 p=0.097	
MHCII tumor	r=0.529 p=0.011	r=0.314 p=0.155	r=0.757 p<0.001	r=0.720 p<0.001	r=0.499 p=0.021

MACROPHAGE- CD45 - CD3

PRIMARY	CD45s	CD45t	CD3s	CD3t
CD68s	r=0.575 p=0.002	r=0.561 p=0.004	r=0.515 p=0.007	r=0.502 p=0.011
CD68t	r=0.735 p<0.001	r=0.807 p<0.001	r=0.735 p<0.001	r=0.812 p<0.001
CD163s	r=0.811 p<0.001	r=0.791 p<0.001	r=0.864 p<0.001	r=0.783 p<0.001
CD163t	r=0.749 p<0.001	r=0.759 p<0.001	r=0.743 p<0.001	r=0.707 p<0.001
CD33s	r=0.601 p=0.002	r=0.602 p=0.002	r=0.629 p=0.001	r=0.631 p=0.001
MHCII tumor	r=0.661 p=0.001	r=0.753 p<0.001	r=0.626 p=0.001	r=0.705 p=0.001

LN	CD45s	CD45t	CD3s	CD3t
CD68s	r=0.410 p=0.047	r=0.564 p=0.006	r=0.448 p=0.028	r=0.633 p=0.002
CD68t	r=0.374 p=0.079	r=0.452 p=0.035	r=0.350 p=0.102	r=0.478 p=0.025
CD163s	r=0.447 p=0.028	r=0.729 p<0.001	r=0.349 p=0.095	r=0.676 p=0.001
CD163t	r=0.359 p=0.092	r=0.738 p<0.001	r=0.210 p=0.335	r=0.573 p=0.005
CD33s	r=0.705 p<0.001	r=0.638 p=0.002	r=0.680 p=0.001	r=0.627 p=0.003
MHCII tumor	r=0.422 p=0.050	r=0.654 p=0.001	r=0.416 p=0.054	r=0.605 p=0.004

very strong, significant correlation<br>r > 0.8<br>p < 0.001	moderate, significant correlation<br>0.4 < r > 0.6<br>p < 0.05
strong, significant correlation<br>r > 0.6<br>p < 0.01	weak, insignificant correlation<br>r < 0.4<br>p > 0.05

Table S2. Cut-off values for macrophage related IHC biomarkers (HIGH- and LOW tumor categories)
Percentages represent the ratios of HIGH- and LOW tumors from the whole patient cohort.

	CD68s	CD68t	CD163s	CD163t	MHCII immune	MHCII tumor	CD33s	NE
**PRIMARY**								
HIGH	13/29 (45%)	10/29 (34%)	20/29 (69%)	8/29 (28%)	15/29 (52%)	10/29 (34%)	12/29 (41%)	19/29 (66%)
LOW	16/29 (55%)	19/29 (66%)	9/29 (31%)	21/29 (72%)	14/29 (48%)	19/29 (66%)	17/29 (59%)	10/29 (34%)
cut-off (cell/mm^2) score (0-3)	291.23	39.68	413.33	63.65	0-1 --> LOW<br>2-3 --> HIGH	0-1 --> LOW<br>2-3 --> HIGH	0-1 --> LOW<br>2-3 --> HIGH	
**LN**								
HIGH	10/29 (34%)	5/29 (17%)	13/29 (45%)	7/29 (24%)	16/29 (55%)	10/29 (34%)	10/29 (34%)	22/29 (76%)
LOW	19/29 (66%)	24/29 (83%)	16/29 (55%)	22/29 (76%)	13/29 (45%)	19/29 (66%)	19/29 (66%)	7/29 (24%)
cut-off (cell/mm^2) score (0-3)	631.25	115.48	246.94	27.82	0-1 --> LOW<br>2-3 --> HIGH	0-1 --> LOW<br>2-3 --> HIGH	0-1 --> LOW<br>2-3 --> HIGH	

Table S3. Cut-off values for IHC biomarkers CD45 and CD3 (HIGH- and LOW tumor categories)
Percentages represent the ratios of HIGH- and LOW tumors from the whole patient cohort.

	**CD45s**	**CD45t**	**CD3s**	**CD3t**
**PRIMARY**				
HIGH	15/28 (54%)	13/28 (46%)	11/28 (39%)	11/28 (39%)
LOW	13/28 (46%)	15/28 (54%)	17/28 (61%)	17/28 (61%)
cut-off (cell/mm^2) score (0-3)	568.93	45.51	201.55	15.96
**LN**				
HIGH	20/28 (69%)	8/29 (28%)	18/29 (62%)	5/29 (17%)
LOW	9/29 (31%)	21/29 (72%)	11/29 (38%)	24/29 (83%)
cut-off (cell/mm^2) score (0-3)	848.98	86.08	298.48	43.24

Table S4. HIGH and LOW annotation for NE-high and NE-low tumors according to protein marker expression

	CD68		CD163		MHCII			CD45		CD3	
**PRIMARY**	CD68s	CD68t	CD163s	CD163t	MHCII immune	MHCII tumor	CD33s	CD45s	CD45t	CD3s	CD3t
**12-I**	HIGH	LOW	LOW	LOW	LOW	LOW	LOW	HIGH	LOW	LOW	LOW
**6-I**	HIGH	HIGH	HIGH	HIGH	HIGH	HIGH	LOW	HIGH	HIGH	HIGH	HIGH
**21-I**	HIGH	HIGH	HIGH	HIGH	HIGH	HIGH	HIGH	HIGH	LOW	HIGH	LOW
**26-I**	HIGH	HIGH	HIGH	HIGH	HIGH	HIGH	HIGH	HIGH	NA	NA	NA
**36-I**	NA	NA	NA	NA	NA	NA	NA	NA	NA	NA	NA
**39-I**	HIGH	HIGH	HIGH	HIGH	HIGH	HIGH	NA	HIGH	HIGH	HIGH	HIGH
**355-I**	LOW	LOW	HIGH	LOW	HIGH	LOW	LOW	LOW	LOW	LOW	LOW
**346-I**	LOW	LOW	HIGH	LOW	LOW	LOW	HIGH	LOW	LOW	LOW	LOW
**270-I**	LOW	LOW	HIGH	HIGH	HIGH	HIGH	LOW	HIGH	HIGH	HIGH	HIGH
**209-I**	HIGH	LOW	HIGH	LOW	HIGH	HIGH	HIGH	HIGH	HIGH	HIGH	HIGH
**190-I**	HIGH	HIGH	HIGH	HIGH	HIGH	HIGH	HIGH	HIGH	HIGH	HIGH	HIGH
**134-I**	LOW	HIGH	HIGH	LOW	HIGH	LOW	HIGH	LOW	HIGH	HIGH	HIGH
**104-I**	LOW	LOW	LOW	LOW	LOW	LOW	LOW	LOW	LOW	LOW	LOW
**126-I**	LOW	LOW	LOW	LOW	LOW	LOW	LOW	LOW	LOW	LOW	LOW
**132-I**	LOW	LOW	HIGH	LOW	LOW	LOW	LOW	HIGH	HIGH	LOW	HIGH
**148-I**	LOW	LOW	LOW	LOW	LOW	LOW	LOW	LOW	LOW	LOW	LOW
**171-I**	LOW	LOW	HIGH	LOW	NA	NA	HIGH	HIGH	HIGH	HIGH	LOW
**188-I**	LOW	LOW	HIGH	LOW	LOW	LOW	NA	LOW	LOW	LOW	LOW
**204-I**	LOW	LOW	HIGH	LOW	NA	NA	NA	HIGH	HIGH	LOW	HIGH
**210-I**	LOW	LOW	HIGH	LOW	NA	NA	NA	HIGH	HIGH	LOW	HIGH
**216-I**	LOW	LOW	LOW	LOW	LOW	LOW	LOW	LOW	LOW	LOW	LOW
**268-I**	LOW	LOW	HIGH	LOW	HIGH	LOW	HIGH	LOW	LOW	LOW	LOW
**271-I**	LOW	LOW	LOW	LOW	HIGH	LOW	LOW	LOW	LOW	LOW	LOW
**28-I**	HIGH	HIGH	HIGH	HIGH	HIGH	LOW	LOW	HIGH	HIGH	HIGH	HIGH
**29-I**	HIGH	LOW	LOW	LOW	NA	NA	LOW	LOW	LOW	LOW	LOW
**332-I**	LOW	LOW	HIGH	HIGH	HIGH	HIGH	LOW	HIGH	LOW	LOW	LOW
**33-I**	HIGH	HIGH	HIGH	LOW	HIGH	HIGH	HIGH	HIGH	HIGH	HIGH	HIGH
**352-I**	HIGH	HIGH	LOW	LOW	HIGH	HIGH	LOW	HIGH	HIGH	LOW	LOW
**354-I**	LOW	LOW	LOW	LOW	LOW	LOW	LOW	LOW	LOW	LOW	LOW
**363-I**	LOW	LOW	HIGH	LOW	HIGH	LOW	HIGH	HIGH	LOW	HIGH	LOW
**3-I**	HIGH	LOW	HIGH	LOW	NA	NA	LOW	LOW	LOW	LOW	LOW
**41-I**	LOW	LOW	HIGH	LOW	HIGH	LOW	HIGH	LOW	LOW	LOW	LOW
**LN**											
**12-III**	HIGH	HIGH	HIGH	LOW	HIGH	LOW	LOW	HIGH	LOW	HIGH	LOW
**33-III**	LOW	LOW	LOW	LOW	NA	NA	NA	HIGH	HIGH	HIGH	HIGH
**36-III**	LOW	LOW	LOW	LOW	LOW	LOW	LOW	HIGH	LOW	HIGH	LOW
**355-III**	HIGH	HIGH	HIGH	HIGH	HIGH	HIGH	HIGH	HIGH	HIGH	HIGH	HIGH
**346-III**	LOW	LOW	LOW	LOW	HIGH	LOW	LOW	LOW	LOW	LOW	LOW
**270-III**	LOW	LOW	HIGH	HIGH	HIGH	LOW	LOW	LOW	LOW	HIGH	LOW
**209-III**	LOW	LOW	HIGH	LOW	HIGH	HIGH	HIGH	HIGH	HIGH	HIGH	LOW
**190-III**	LOW	LOW	HIGH	HIGH	HIGH	HIGH	HIGH	HIGH	HIGH	HIGH	HIGH
**134-III**	LOW	LOW	HIGH	LOW	NA	NA	HIGH	HIGH	LOW	HIGH	LOW
**104-III**	LOW	LOW	LOW	LOW	LOW	LOW	HIGH	HIGH	LOW	HIGH	LOW
**126-III**	LOW	LOW	LOW	LOW	NA	NA	NA	LOW	LOW	LOW	LOW
**132-III**	LOW	LOW	LOW	LOW	LOW	LOW	LOW	HIGH	LOW	HIGH	LOW
**148-III**	LOW	LOW	LOW	LOW	LOW	LOW	LOW	LOW	LOW	LOW	LOW
**171-III**	LOW	LOW	HIGH	HIGH	HIGH	HIGH	LOW	HIGH	HIGH	LOW	LOW
**188-III**	LOW	LOW	LOW	LOW	LOW	LOW	LOW	HIGH	LOW	HIGH	LOW
**204-III**	LOW	LOW	LOW	LOW	NA	NA	LOW	LOW	LOW	LOW	LOW
**210-III**	LOW	LOW	LOW	LOW	LOW	LOW	LOW	LOW	LOW	LOW	LOW
**216-III**	LOW	LOW	LOW	LOW	LOW	LOW	LOW	LOW	LOW	LOW	LOW
**21-III**	HIGH	LOW	HIGH	LOW	HIGH	LOW	LOW	LOW	LOW	HIGH	LOW
**268-III**	LOW	LOW	LOW	LOW	HIGH	LOW	NA	LOW	LOW	LOW	LOW
**26-III**	HIGH	LOW	HIGH	LOW	HIGH	HIGH	HIGH	HIGH	HIGH	HIGH	HIGH
**271-III**	LOW	LOW	LOW	LOW	LOW	LOW	LOW	LOW	LOW	LOW	LOW
**28-III**	HIGH	HIGH	HIGH	HIGH	HIGH	NA	NA	HIGH	HIGH	HIGH	HIGH
**29-III**	HIGH	LOW	HIGH	LOW	HIGH	LOW	HIGH	HIGH	HIGH	HIGH	HIGH
**332-III**	LOW	LOW	LOW	LOW	LOW	LOW	LOW	LOW	LOW	HIGH	LOW
**352-III**	LOW	LOW	LOW	HIGH	HIGH	LOW	LOW	HIGH	HIGH	LOW	LOW
**354-III**	LOW	LOW	LOW	LOW	LOW	LOW	LOW	HIGH	LOW	HIGH	LOW
**363-III**	LOW	LOW	LOW	LOW	LOW	LOW	NA	HIGH	LOW	LOW	LOW
**39-III**	HIGH	LOW	LOW	LOW	HIGH	NA	HIGH	HIGH	LOW	HIGH	LOW
**3-III**	HIGH	LOW	LOW	LOW	LOW	LOW	LOW	HIGH	LOW	HIGH	LOW
**41-III**	LOW	LOW	LOW	LOW	LOW	LOW	LOW	HIGH	LOW	HIGH	LOW
**6-III**	HIGH	HIGH	HIGH	HIGH	HIGH	HIGH	LOW	HIGH	LOW	LOW	LOW

Table S5. Node and edge data for string maps

	Nodes	Edges	edges/node	total edge score (SUM)			Nodes	Edges	edges/node	total edge score (SUM)
**NE-low**	68	148	2.18	94.147	**VS**	**NE-high**	65	77	1.18	51.35
**Immune-oasis**	27	53	1.963	36.98	**VS**	**Immune-desert**	20	21	1.05	13.51
**Immune-oasis NE-high**	32	17	0.531	11.39	**VS**	**Immune-oasis NE-low**	90	133	1.48	90.58
**Immune-oasis NE-high**	37	57	1.54	36.72	**VS**	**Immune-desert NE-high**	23	16	0.69	9.52
**Immune-oasis NE-low**	36	216	6	145.032	**VS**	**Immune-desert NE-low**	24	34	1.42	19.51

Supplemental Table 6. Possible therapeutic interventions on eligible molecular targets.
List of NE-low and NE-high molecular targets against which drugs are already available or under development. *N.A: no available drug*
PDF with links as attachment
