# Supplementary material for: Characterization of Tumor-Associated Macrophages and the Immune Microenvironment in Limited-Stage Neuroendocrine-High and -Low Small Cell Lung Cancer
Source: Biology (Basel). 2021 Jun 4;10(6):502. doi: 10.3390/biology10060502 (PMC8228874; doi:10.3390/biology10060502)
Supplement: Supplementary file 1 [file biology-10-00502-s001.zip › Supplemental Table 6.pdf]

Target	Subcellular localisation	Molecular function	Drug name<br>DrugBank ID	Mechanism<br>Study status	Drug name<br>DrugBank ID	Mechanism<br>Study status
**MMP7**	extracellular	Degrades casein, gelatins of types I, III, IV, and V, and fibronectin.<br>Activates procollagenase	marimastat<br><br>DB00786	MMP-inhibitor<br><br>Phase 3<br>ClinicalTrials	N.A	N.A
**CXCR2**	plasmamembrane	Receptor for interleukin-8 which is a powerful neutrophil chemotactic factor	reparixin<br><br>DB12614	chemokine receptor antagonist<br><br>Phase 3<br>ClinicalTrials	danirixin<br><br>DB11922	chemokine receptor antagonist<br><br>Phase 2<br>ClinicalTrials
**CES1**	cytoplasm	Involved in the detoxification of xenobiotics and in the activation of ester and amide prodrugs	apoptosis- activator-II	CES- inhibitor<br><br>preclinical	avasimibe<br><br>DB06442	ACAT inhibitor<br><br>Phase3<br>PubMed
**ITGB6**	plasmamembrane	Integrin alpha-V:beta-6) is a receptor for fibronectin and cytotactin	integrin-antagonist-1	integrin antagonist<br><br>Phase 1<br>ClinicalTrials	N.A	N.A
**BIRC3**	nucleus	Multi-functional protein which regulates caspases, apoptosis, inflammatory signaling and immunity, mitogenic kinase signaling and cell proliferation.	birinapant<br><br>DB11782	XIAP inhibitor<br><br>Phase 2<br>ClinicalTrials	AT-406	IAP antagonist<br><br>Phase 1<br>ClinicalTrials
**BCAT1**	cytoplasm, mitochondrion	Catalyzes the first reaction in the catabolism of the essential branched chain amino acids leucine, isoleucine, and valine.	L-glutamate<br><br>DB00142	glutamate receptor agonist<br><br>Launched	N.A	N.A
**ALDH2**	Mito-chondrion	This protein is involved the subpathway that synthesizes acetate from ethanol	disulfiram<br><br>DB00822	ALDH-inhibitor<br><br>launched	daidzin<br><br>DB02115	glycosyloxyisoflavone<br><br>Phase 1<br>ClinicalTrials
**TNFSF10**	plasmamebrane, extracellular	Cytokine that induces apoptosis. Its activity may be modulated by binding to the decoy receptors TNFRSF10C/TRAILR3	ONC201<br><br>DB14844	AKT-inhibitor<br><br>Phase 2<br>ClinicalTrials	N.A	N.A
**MYC**	nucleus	Transcription factor that binds DNA in a non-specific manner. Activates the transcription of growth-related genes, promoting VEGFA production and subsequent angiogenesis	AZD5153	bromodomain inhibitor<br><br>Phase1<br>ClinicalTrials	mivebresib	bromodomain inhibitor<br><br>Phase 1<br>ClinicalTrials

**CDH2**	plasmamembrane	Calcium-dependent cell adhesion protein. Acts as a regulator of neural stem cells quiescence by mediating anchorage of neural stem cells	exherin	cadherin antagonist<br>Phase 2<br>ClinicalTrials	N.A	N.A
**EGLN2**	nucleus	Cellular oxygen sensor that catalyzes, under normoxic conditions, the post-translational formation of 4-hydroxyproline in hypoxia-inducible factor (HIF) alpha proteins	FG-2216<br><br>DB08687	hypoxia inducible factor prolyl hydroxylase inhibitor<br><br>Phase 2<br>ClinicalTrials	vadadustat<br><br>DB12255	hypoxia inducible factor prolyl hydroxylase inhibitor<br><br>Phase 3<br>ClinicalTrials
**PAK7**	nucleus, mitochondrion	Serine/threonine protein kinase that plays a role in a variety of different signaling pathways including cytoskeleton regulation, cell migration, proliferation or cell survival	GSK690693<br><br>DB12745	AKT inhibitor<br><br>Phase 1<br>ClinicalTrials	N.A	N.A
**TPO**	plasma-mebrane, extracellular	Iodination and coupling of the hormonogenic tyrosines in thyroglobulin to yield the thyroid hormones T_3 and T_4	carbimazole<br><br>DB00389	antithyroid agent<br><br>Launched	propylthiouracil<br><br>DB00550	thyroid peroxidase inhibitor<br><br>Launched
